# The role of cell-envelope synthesis for envelope growth and cytoplasmic density in *Bacillus subtilis*

**DOI:** 10.1093/pnasnexus/pgac134

**Published:** 2022-07-26

**Authors:** Yuki Kitahara, Enno R Oldewurtel, Sean Wilson, Yingjie Sun, Silvia Altabe, Diego de Mendoza, Ethan C Garner, Sven van Teeffelen

**Affiliations:** Département de Microbiologie, Infectiologie, et Immunologie, Faculté de Médecine, Université de Montréal, Montréal, QC, Canada; Université de Paris, Paris, France; Microbial Morphogenesis and Growth Lab, Institut Pasteur, Paris, France; Microbial Morphogenesis and Growth Lab, Institut Pasteur, Paris, France; Department of Molecular and Cellular Biology, Harvard University, Cambridge, USA; Center for Systems Biology, Harvard University, Cambridge, MA, USA; Department of Molecular and Cellular Biology, Harvard University, Cambridge, USA; Center for Systems Biology, Harvard University, Cambridge, MA, USA; Instituto de Biología Molecular y Celular de Rosario (IBR)-Conicet- and Departamento de Microbiología, Facultad de Ciencias Bioquímicas y Farmacéuticas, Universidad Nacional de Rosario, Rosario, Argentina; Instituto de Biología Molecular y Celular de Rosario (IBR)-Conicet- and Departamento de Microbiología, Facultad de Ciencias Bioquímicas y Farmacéuticas, Universidad Nacional de Rosario, Rosario, Argentina; Department of Molecular and Cellular Biology, Harvard University, Cambridge, USA; Center for Systems Biology, Harvard University, Cambridge, MA, USA; Département de Microbiologie, Infectiologie, et Immunologie, Faculté de Médecine, Université de Montréal, Montréal, QC, Canada; Microbial Morphogenesis and Growth Lab, Institut Pasteur, Paris, France

## Abstract

All cells must increase their volumes in response to biomass growth to maintain intracellular mass density within physiologically permissive bounds. Here, we investigate the regulation of volume growth in the Gram-positive bacterium *Bacillus subtilis*. To increase volume, bacteria enzymatically expand their cell envelopes and insert new envelope material. First, we demonstrate that cell-volume growth is determined indirectly, by expanding their envelopes in proportion to mass growth, similarly to the Gram-negative *Escherichia coli*, despite their fundamentally different envelope structures. Next, we studied, which pathways might be responsible for robust surface-to-mass coupling: We found that both peptidoglycan synthesis and membrane synthesis are required for proper surface-to-mass coupling. However, surprisingly, neither pathway is solely rate-limiting, contrary to wide-spread belief, since envelope growth continues at a reduced rate upon complete inhibition of either process. To arrest cell-envelope growth completely, the simultaneous inhibition of both envelope-synthesis processes is required. Thus, we suggest that multiple envelope-synthesis pathways collectively confer an important aspect of volume regulation, the coordination between surface growth, and biomass growth.

Significance Statement Bacterial cell volume determines the intracellular density of macromolecules and is essential for intracellular organization. Therefore, biomass growth and cell-volume growth must be coordinated. Volume growth, in turn, is governed by the enzymatic expansion of the cell envelope. Gram-positive bacteria are surrounded by a plasma membrane and a thick peptidoglycan cell wall. While much emphasis in the past has been placed on the role of cell-wall synthesis for cell-envelope growth, we demonstrate here that cell-wall insertion and membrane synthesis are equally required for the coordination of cell-envelope growth and biomass growth. However, neither pathway is solely rate-limiting. These findings will guide future research on the molecular regulation of envelope growth.

## Introduction

In bacteria and other organisms, the cytoplasm is crowded with macromolecules, notably protein, RNA, and DNA, which occupy about 20% to 40% of the volume (1, 2). Cytoplasmic crowding is important for cell physiology as it directly impacts macromolecular diffusion (3,4), molecular interactions (5), and chromosome organization (6). Furthermore, it was also suggested that crowding maximizes biomass growth rate (7, 8). To maintain the density of macromolecules and other cytoplasmic components within physiologically permissive bounds or to achieve optimal crowding, cells must coordinate their volume growth rate with the rate of biomass growth.

We recently studied this problem in the Gram-negative bacterium *Escherichia coli* (9). By measuring single-cell dry mass and cell dimensions independently using quantitative phase microscopy, we showed that cell-volume growth is determined indirectly on the timescale of about one generation: Cells increase their surface area rather than volume in proportion to dry-mass growth. Thus, they maintain a constant ratio of cell-surface area *S* to total cellular dry mass *M*.

During steady-state growth, when cell width remains almost constant, this coupling guarantees that cell volume grows roughly in proportion to mass, because surface area, volume, and length increase approximately in proportion to one another. However, if cells systematically increase their width, for example after a nutrient upshift, cell volume grows faster than surface area. Thus, the cytoplasm is diluted, and dry-mass density drops (9).

The robust coupling of surface area and dry mass in *E. coli* implies that surface area increases by the same relative amount as dry mass, independently of dry-mass density, turgor pressure, or instantaneous growth rate. The surface-to-mass coupling might, thus be metabolic in nature, through the production of a rate-limiting cell-envelope component, while other physiological processes such as crowding and turgor pressure have no apparent influence on surface growth on short timescales, in agreement with previous work by Rojas et al. (10). However, the metabolic pathways responsible for surface-to-mass coupling remain to be identified in *E. coli* or any other bacterium.

Here, we investigate how the Gram-positive bacterium *Bacillus subtilis* coordinates volume and biomass growth. Gram-negative and Gram-positive bacteria differ in envelope structure and envelope growth in fundamental ways: Gram-negative bacteria are surrounded by a thin peptidoglycan cell wall and by a mechanically important outer membrane. On the contrary, Gram-positive bacteria lack an outer membrane but are surrounded by a much thicker cell wall. Furthermore, osmotic pressure (turgor) has an influential role in surface-area expansion in *B. subtilis* but not in *E. coli*. More specifically, *B. subtilis* changes its rate of surface growth in response to changes of turgor pressure (11), while *E. coli* does not (10). It, thus remains unclear whether the robust surface-to-mass coupling observed in *E. coli* (9) is maintained in *B. subtilis*. Furthermore, the role of the insertion of peptidoglycan and other envelope components for surface growth remains to be explored.

Using quantitative phase microscopy, we demonstrate here that surface and mass are robustly coupled during growth of *B. subtilis*, even if cell width and, therefore, dry-mass density changes. Specifically, dry-mass density is inversely proportional to width at the single-cell level. Furthermore, we observed similar correlations at the population level when systematically varying cell width by modulating the expression of class-A penicillin binding proteins (aPBPs) as previously described (12). Upon increase of cell width, dry-mass-density decreases by up to 30%, but biomass growth rate and cell-wall insertion remain remarkably constant. Thus, dry-mass density and crowding do not govern surface growth.

Which pathway is responsible for the coupling between surface and dry-mass growth? Physically, cell-surface area is governed by the peptidoglycan cell wall. Thus, cell-wall cleaving autolysins are strictly required for growth. In visionary and influential work, Koch suggested that “smart autolysins” are activated based on mechanical stress in the cell wall (13), which, in turn, is caused by turgor pressure. However, more recent works imply that the MreB-linked cell-wall insertion machinery provides the major regulator of cell elongation in *B. subtilis* (11, 14–17). A regulatory role of peptidoglycan insertion for autolytic activity is supported by previous studies suggesting that the two redundantly essential cell-wall hydrolases of *B. subtilis*, LytE and CwlO, are controlled by the three MreB homologs (16, 18). Furthermore, the amount of moving MreB filaments and cell-envelope growth are highly correlated across different growth conditions (17), which is compatible with a rate-limiting role of MreB-based cell-wall insertion. However, a molecular mechanism linking cell-wall insertion and cell-wall expansion has not been identified. Furthermore, there is also evidence against this hypothesis: specifically, sublethal concentrations of cell-wall antibiotics such as fosfomycin do not affect cell-elongation rate (11). Furthermore, we recently discovered that peptidoglycan insertion is not rate-limiting in *E. coli* (19), contrary to wide-spread belief (20). Thus, the connection between cell-wall insertion, biomass growth, and surface expansion remains unclear.

Another essential envelope component is the cytoplasmic membrane. Previously, Rojas et al. provided combined experimental and model-based evidence that membrane tension is important to facilitate cell-wall insertion, which together with turgor pressure, might be responsible for driving cell-wall expansion (11). Furthermore, Müller et al. and Zielińska et al. demonstrated that membrane fluidity and membrane microdomain organization affects cell-wall insertion (21, 22). Interestingly, inhibition of membrane synthesis through glycerol starvation in a glycerol auxotroph increases buoyant mass density (23), which is compatible with the idea that surface area increases more slowly than mass during the arrest of membrane synthesis. Whether membrane insertion constitutes a direct link between cell-surface area and biomass growth remains to be investigated.

In this work, we demonstrate that both cell-wall insertion and cell-membrane insertion are required for proper surface growth and for the maintenance of *S*/*M*. If either of the two processes is inhibited, surface growth is severely reduced, while biomass growth continues. Interestingly, though, cell-wall insertion is not directly coupled to cell-surface growth. Instead, we observe a delay between the arrest of peptidoglycan insertion and the reduction of surface growth, in agreement with previous observations (24). Furthermore, surface growth continues at a reduced rate, even though cell-wall insertion is inhibited. Similarly, cells can reduce surface growth even if the rate of peptidoglycan insertion remains high. Thus, cell-wall insertion is important but not rate-determining for cell-surface growth. Similarly, we find that membrane insertion is required for proper surface growth, but the visible overproduction of membrane does not lead to increased surface growth. Once the perturbation of envelope synthesis is relieved, *S*/*M* returns rapidly to its pretreatment value. Finally, we demonstrate that the combined inhibition of multiple pathways of cell-envelope synthesis can reduce surface growth by a similar relative amount as seen during glucose starvation. On the contrary, changes of turgor pressure are not responsible for this phenotype.

Together, our experiments demonstrate that cell-volume growth is determined indirectly, by coupling surface growth to mass growth, with an important role of different envelope-synthesis pathways for cell-surface growth.

## Results

### Cells grow surface rather than volume in proportion to biomass

To study the relationship between cell-volume growth and biomass growth in *B. subtilis*, we quantified single-cell dimensions and dry mass of live cells in absolute terms using quantitative phase microscopy, similarly to our recent measurements on *E. coli* (9). However, because the cell wall is a thick and uneven layer in Gram-positive bacteria, we decided to concentrate on cytoplasmic properties rather than whole-cell properties. Specifically, we calculated cytoplasmic volume *V*, surface area *S*, and width *W* based on 2D cell contours from phase-contrast images using the morphometrics tool (25), after calibration based on the membrane dye FM 4-64. To obtain cytoplasmic mass, we first measured the total cellular dry mass *M*_tot_ using Spatial Light Interference Microscopy (SLIM) (26), a variant of quantitative phase microscopy, as demonstrated (9). Subsequently, to obtain cytoplasmic mass *M*, we subtracted a constant fraction of 14%, which represents the dry mass of the cell wall obtained by bulk measurements (two biological replicates: 13.8% and 14.2%). Other extracytoplasmic contributions to total mass, for example from periplasmic proteins, are small (< 5%) and allocated to the cytoplasmic mass for simplicity (“Materials and Methods”).

**Fig. 1.**
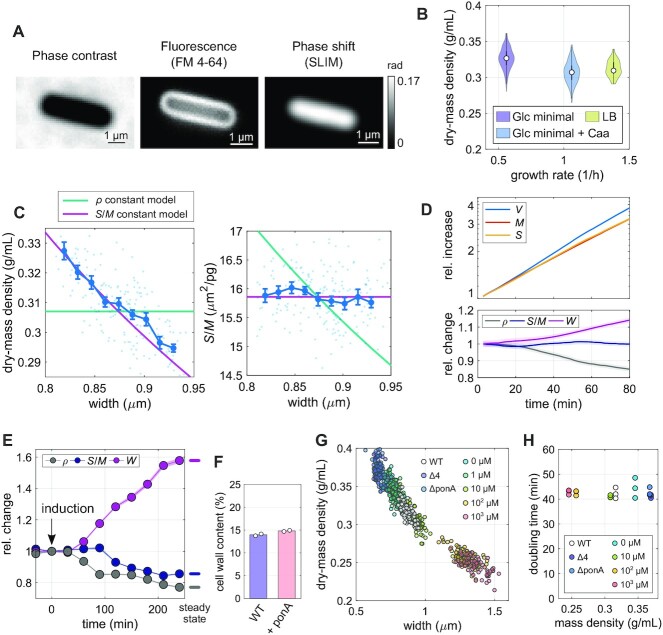
Volume growth of *B. subtils* is determined indirectly through the coupling of surface growth and dry-mass growth. (A) Snapshots of a wild-type cell labeled with the membrane dye FM4-64, in minimal medium with glucose and casamino acids (S7_50_+GlcCaa) taken by phase-contrast microscopy, fluorescence microscopy, and SLIM (grayscale bar: phase shift). (B) Distribution of single-cell dry-mass density of wild-type cells grown to exponential phase in S7_50_+Glc medium, S7_50_+GlcCaa medium, or in LB (white circles = median; gray rectangles = interquartile range). (C) Width dependency of dry-mass density and surface-to-mass ratio in S7_50_+GlcCaa medium (dots: single-cell measurements, lines: binned averages ± SE (blue), model prediction for spherocylinder with constant surface-to-mass ratio (pink), or constant dry-mass density (green). (D)  Single-cell time lapse of filamenting bMD834 cells during *ponA* induction on agarose pad (S7_50_+GlcCaa containing 1 mM IPTG and 30 mM xylose). Relative increase (top) of volume, surface, and dry mass. Bottom: relative change of width, surface-to-mass ratio, and dry-mass density (solid lines + shadings = average ± 2*SE). (E)  Relative change of average width, surface-to-mass ratio, and dry-mass density (average ± SE) of bMD586 cells upon *ponA* induction (1 mM IPTG at time = 0) in batch culture (S7_50_+GlcCaa). Points represent averages from single-cell snapshots. Culture was back diluted to keep OD600 < 0.3. (F) Cell-wall content per cellular dry mass of wild-type and bMD586 (1 mM IPTG) cells grown in S7_50_+GlcCaa (white circles = biological replicates). (G) Correlation between mass density and width under different expression levels of aPBPs obtained from wild-type, bSW164 (Δ4), bKY42 (Δ*ponA*), and bMD586 cells during steady-state growth in S750+GlcCaa. To titrate PonA expression level in bMD586, IPTG was added from 1 to 1,000 *μ*M (see Figure S1D to compare cell width, ρ, and *S*/*M* of different conditions.). (H) Comparison between average mass density (obtained from OD600 between 0.02 and 0.3) and bulk doubling time of the same cultures as in G (circles = biological replicates). See Table S1 for the number of analyzed cells, the absolute values used for normalization, and information on replicates.

First, we grew cells to exponential phase in different growth media in batch and took snapshots on agarose pads (Fig. 1A). Despite almost 3-fold differences in average growth rate, the average cytoplasmic dry-mass density of about 0.31 to 0.33 g/ml (Fig. 1B) varies by no more than 5% between conditions, in agreement with independent refractive-index measurements through immersive refractometry (Figure S1A). Furthermore, single-cell variations in dry-mass density are remarkably small (≈ 3% to 5%). Interestingly, similar absolute values and variations were measured in *E. coli* (9).

In *E. coli*, we previously observed that cells control dry-mass density indirectly, by increasing surface area rather than volume in proportion to mass (9). Dry-mass density ρ = *M*/*V* can be expanded as the ratio of surface-to-volume and surface-to-mass ratios, ρ = (*S*/*V*)/(*S*/*M*). For spherocylindrical cells, *S*/*V* scales approximately inversely with width *W* according to *S*/*V* ≈ 4/*W*. We can, thus write ρ ≈ 4/[*W*(*S*/*M*)]. If cells grew surface area in proportion to mass just like *E. coli*, independently of any change of dry-mass density, we would expect that dry-mass density is inversely proportional to cell width, while the surface-to-mass ratio *S*/*M* shows no or weak dependency on width. Investigating correlations between single-cell values of dry-mass density, *S*/*M*, and width, we found that this is indeed the case (Fig. 1C). We observed the same behavior in a different growth medium (Figure S1B). Our observations thus suggest that *B. subtilis* controls cell volume indirectly, by growing surface area rather than volume in proportion to mass, just like the Gram-negative *E. coli* (9).

To study how stably the surface-to-mass ratio is maintained over time, we also conducted time-lapse microscopy experiments. To study cells for more than one generation of growth, we inhibited cell division by inducing the expression of MciZ, a peptide that inhibits Z-ring formation (27), about one doubling time prior to microscopy using strain bAB56 (*mciZ*::spec-pHyperSpank-*mciZ*). MciZ is induced from a xylose- or IPTG-inducible promoter in all subsequent time-lapse experiments unless performed on wild-type cells (Table S1).

Single-cell values of *S*/*M* and width remain nearly constant during one mass doubling (Figure S1C). Accordingly, mass density remains also nearly constant on this timescale (Figure S1C).

### Modulation of cell width through class-A PBPs changes average dry-mass density without perturbing growth rate

Next, we tested whether the surface-to-mass coupling was also maintained during systematic changes of cell width. It was previously described that cell width changes in response to the balance between the activities of two different peptidoglycan-synthesizing machineries, the MreB-actin–rod complex and the aPBPs (12). We, thus modulated the expression level of the major aPBP PonA by inducing expression from an IPTG-inducible promoter using strain bMD834 (*yhdG*::cat-pHyperSpank-*ponA*, *ponA*::kan, *yvbJ*::erm-pXyl-*mciZ*, (12); Fig. 1D).

Specifically, we took time-lapse movies of filamentous cells after *ponA* induction. Throughout the time lapse of nearly two mass-doubling times, 〈*S*/*M*〉 remained nearly constant while width increased. Accordingly, mass density decreased by about 20%. This result confirms that the surface-to-mass ratio is controlled independently of cell width. To study the behavior at longer times, we also conducted the *ponA* induction experiment using strain bMD586 (*yhdG*::cat-pHyperSpank-*ponA*, *ponA*::kan, (12)) in batch culture and took snapshots of cells at regular intervals (Fig. 1E). After about two mass-doubling times, 〈*S*/*M*〉 started to decrease toward a new steady-state value. However, since relative variations in 〈*S*/*M*〉 are about 3-fold smaller than relative variations in width, the final mass-density is still reduced by more than 20% (according to the relationship ρ ≈ 4/*W*/(*S*/*M*)).

Previously, Dion et al. showed that the cell wall is thicker upon high PonA expression than in wild-type cells (12), suggesting that the amount of cell-wall material per surface area is increased. We, thus speculated that the decrease of 〈*S*/*M*〉 might be a consequence of the thicker cell wall, while the rate of cell-wall insertion per mass and, thus the amount of cell-wall material per mass remains unchanged (for the description of a simple model see “Materials and Methods”). Indeed, we observed that the total amount of cell-wall material per biomass remains constant upon PonA overexpression (Fig. 1F). The reduction of *S*/*M* is, therefore, not a consequence of reduced cell-wall synthesis, but a consequence of cell-wall thickening. We will come back to the role of cell-wall insertion in the next section.

To investigate the dependency of mass density on width over a broad range of average widths, we modulated *ponA* expression using different inducer concentrations and additionally used mutants that lack either PonA (Δ*ponA*;bKY42) or all four known aPBPs (Δ4; bSW164). We found that dry-mass density showed a monotonic inverse correlation with cell width (Fig. 1G; Figure S1D), both at the population level and at the single-cell level. Deviations from wild-type density were as high as ± 20%. We confirmed these changes through immersive refractometry (Figure S1E).

Previous theoretical work suggests that mass growth rate might depend on intracellular density and crowding (7,8). However, we found that growth rate remains constant across the broad range of mass densities observed here (Fig. 1H). Previous reports of reduced bulk growth rate of aPBP mutants measured at 37°C (28) was subsequently explained to partial cell lysis, while single-cell growth rate remains high (12,29). However, we did not observe lysis, likely due to the reduced temperature and poorer growth medium.

In conclusion, dry-mass density decreases with increasing cell width, both at the single-cell and at the population level, without affecting biomass growth rate.

### Surface expansion and mass growth are robustly coupled during nutrient shifts

**Fig. 2.**
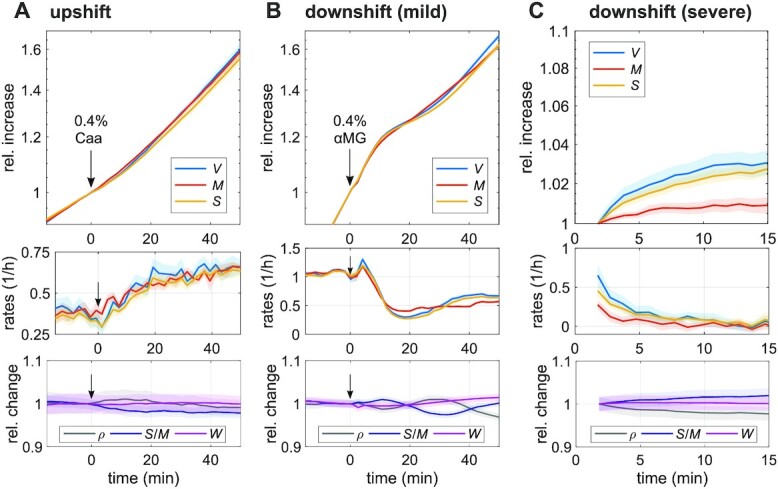
Surface growth is well-coupled during nutrient shifts. (A) Single-cell time lapse of filamentous cells (bAB56) on agarose pad during nutrient upshift from S7_50_+Glc to S7_50_+GlcCaa at time = 0 min (by addition of casamino acids to the top of the agarose pad). Relative increase (top) and rates (mid) of volume, surface, and dry mass. Bottom: relative change of average dry-mass density, surface-to-mass ratio, and width (solid lines + shadings = average ± 2*SE). (B) Single-cell time lapse of filamentous cells (bAB56) during mild nutrient downshift from S7_50_+GlcCaa to S7_50_+GlcCaa + 0.4% glucose analog (αMG). Otherwise the same as in (A). (C) Single-cell time lapse of wild-type cells during severe nutrient downshift. At time = 0, the wild-type cells growing in S7_50_+GlcCaa were put on agarose pad (S7_50_ + 2% αMG, without nutrients). We took time-lapse movie every 1 min. Otherwise the same as in (A).

In *E. coli*, we previously observed that the surface-to-mass ratio is maintained nearly constant during rapid changes of growth rate in nutrient shifts, apart from transient variations ascribed to changes of turgor pressure (9). To test the ability of *B. subtilis* to respond to changes of mass growth rate, we studied single cells in time-lapse microscopy experiments on agarose pads during nutrient shifts (Fig. 2A–C).

For a nutrient upshift we grew cells in minimal medium supplemented with glucose (S7_50_+Glc) and then added casamino acids in form of a droplet at time *t* = 0 (Fig. 2A). Biomass growth rate increased by 60% over the course of about 20 min. During this period, surface growth increased nearly synchronously, thus maintaining *S*/*M* nearly constant, both in terms of the population average (Fig. 2A) and at the single-cell level (Figure S2A). Since width remains almost constant, mass density also remains nearly constant.

We also measured the behavior during a nutrient downshift. To that end we exposed cells growing on minimal medium supplemented with glucose and casamino acids (S7_50_+GlcCaa) to 0.4% alpha methylglucoside (alpha-MG), a nonmetabolizable analog of glucose (30). Within 10 min after adding the droplet of alpha-MG, growth rates of mass, surface, and volume drop by more than 2-fold (Fig. 2B). The beginning of the drop of all rates is nearly synchronous, with a delay of surface growth rate of no more than 2 min. Subsequently, surface and volume growth rates undershoot by about 30% and then oscillate around the constant mass growth rate. Growth-rate variations only cause minor deterministic variations of *S*/*M* and mass density (of about 2%), which are also observed at the single-cell level (Figure S2B). We will come back to the cause of the oscillations in the “Discussion.”

To study a potential delay between surface and mass growth at a more minute scale, we conducted a more severe downshift (using a higher alpha-MG concentration and embedding the drug in the pad prior to microscopy). This shift, which can be regarded as starvation for glucose, leads to a reduction of mass-growth rate by about 90% within 3 min (Fig. 2C; Figure S2C). Surface growth rate drops by about 3-fold within the same duration and then approaches complete arrest on the timescale of 5 min.

In conclusion, surface growth responds to changes of mass growth rate on the timescale of minutes. Deviations between surface- and mass-growth rate during and after nutrient shifts are small enough that 〈*S*/*M*〉 and mass density remain almost constant.

### Inhibition of peptidoglycan insertion decouples surface growth from biomass growth

Next, we wondered how surface growth is coupled to biomass growth mechanistically. Since cells modulate surface-growth rate rapidly after nutrient shifts (within minutes) we focused our investigation on processes that can undergo rapid changes, notably envelope metabolism and turgor pressure, while we ignore slower processes of gene regulation, which might have important roles on long timescales but are likely not responsible for the initial response.

First, we wondered whether a drop in turgor pressure might be responsible for the rapid reduction of envelope growth observed during a nutrient downshift (Fig. 2C). We previously observed that nutrient shifts lead to changes of turgor in *E. coli*. Furthermore, hyperosmotic shocks cause a reduction of elongation rate in *B. subtilis* (11) but not in *E. coli*. A reduction of elongation rate in *B. subtilis* by about 3-fold was previously shown to require a hyperosmotic shock of about 1 Osm (11). Notably, the reduction in elongation rate reported there (over many minutes) cannot be the result of a simple elastic shrinkage of the cell wall but must be due to a change of the rate of plastic cell-wall expansion, which we refer to as envelope growth or surface growth. Here, we observed that hyperosmotic shocks cause a dose-dependent elastic reduction of cell width (Figure S3A). However, during our nutrient downshifts we did not observe a change of width (Fig. 2B and C; Figure S2B and S2C). We can, therefore, rule out that a drop of turgor is responsible for the rapid reduction of elongation rate.

Next, we tested the role of cell-wall insertion. Cell-wall insertion is generally thought to limit surface growth (11,14,31). To test the potentially rate-limiting role of peptidoglycan synthesis for surface growth, we first inhibited cell-wall insertion by treating cells with the antibiotic vancomycin, which binds to the D-Ala-D-Ala terminus of peptidoglycan precursor molecules and thus inhibits cross-linking of new peptidoglycan material (32). To monitor single-cell growth, we studied cells during time-lapse microscopy and added the drug to the pad about 30 min after placing cells on the microscope, similar to our nutrient-shift experiments (Fig. 2A and B).

**Fig. 3.**
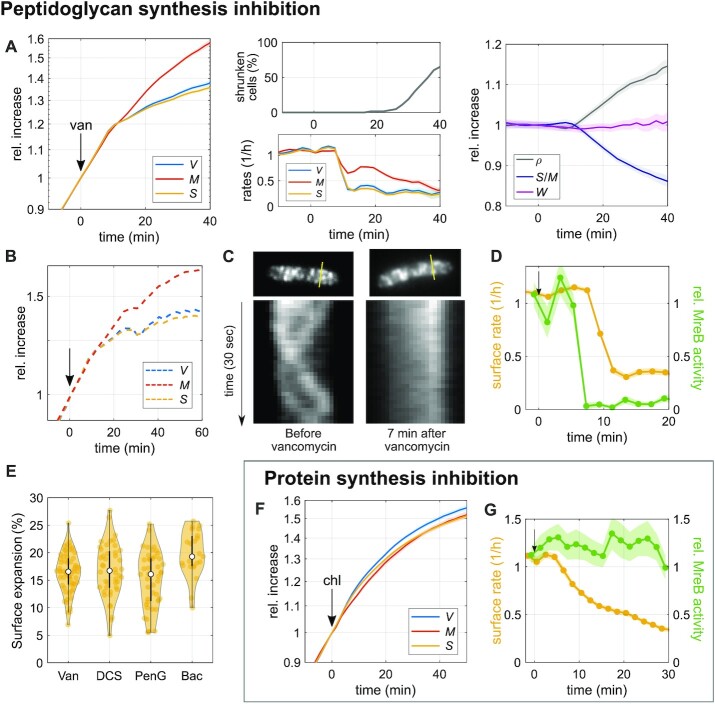
Inhibition of peptidoglycan insertion decouples surface growth from biomass growth. (A) and (B) Single-cell time lapse of filamenting cells (bAB56) grown in S7_50_+GlcCaa medium and treated with vancomycin (50 *μ*g/ml), which was added on top of the agarose pad at time = 0. Relative increase (left) and rates (middle-bottom) of volume, surface, and dry mass. After 20 min, a fraction of cells starts to shrink in surface area (middle-top) and lose part of their mass (see also Figure S3C). Right: relative change of dry-mass density, surface-to-mass ratio, and width (solid lines + shadings = average ± 2*SE). (B) Relative increase of volume, surface, and dry mass for a representative single cell. (C) Kymographs of MreB-GFP rotation in bYS19 cells during 30 s movie before and 7 min after vancomycin addition (as in A) along the lines indicated in MreB-GFP snapshots (top). (D) Comparison of surface expansion rate (yellow; as in A) and relative MreB activity (total length of MreB tracks divided by projected cell area and movie duration; green line + shading = average ± SE) of vancomycin-treated cells to that of nonperturbed cells. (E) Residual surface expansion after MreB motion was arrested by Van (vancomycin 50 *μ*g/ml), DCS (D-cycloserine 10 mM), PenG (Penicillin G 0.5 mg/ml), and Bac (Bacitracin 0.5 mg/ml). Experiments were performed in the same way as (A) and (C) (yellow dots = single-cell values; white circles = median; and gray rectangles = interquartile range). (F) Single-cell time lapse of filamenting cells (bAB56) treated with chloramphenicol (100 *μ*g/ml). Otherwise the same as in A. (G) Comparison of surface expansion rate (yellow: as in F) and relative MreB activity (green line + shading = average ± SE) of chloramphenicol-treated cells to that of nonperturbed cells.

Drug treatment leads to a sudden reduction of surface expansion about 10 min after adding the drug, while mass growth is affected much less (Fig. 3A). The same behavior is observed at the single-cell level (Figure S3B). The 10-min delay between drug addition and reduction of growth rates is at least partially due to the time it takes for the drug to diffuse through the agarose pad (see also next section). Accordingly, *S*/*M* decreases and biomass density increases in inverse proportion to *S*/*M* (Fig. 3A). See Figure S3B for single-cell traces.

About 10 min after the reduction of surface growth rate, some cells shrink in surface area and volume (Fig. 3B), demonstrating a transient loss of envelope integrity and osmotic pressure. Interestingly, though, many cells continue to grow after such events (Figure S3C; Video S1).

We observed the same qualitative behavior when targeting cell-wall synthesis with different drugs that inhibit peptidoglycan-precursor synthesis (D-cycloserine), precursor transport (bacitracin), or cell-wall cross-linking (penicillin G; Figure S3D). Thus, proper cell-wall insertion is apparently required for the maintenance of *S*/*M* during growth.

### Cell-envelope expansion can proceed in the absence of cell-wall insertion or protein expression

While cell-wall insertion is apparently required for the coordination between surface and biomass growth, cells still continue growing in surface area after drug treatment, even if at a reduced rate (Fig. 3A; Figure S3B–S3D). To demonstrate that cell-wall insertion was indeed arrested after drug treatment, we tracked the movement of MreB-actin filaments of the mutant strain (bYS19) that expresses a GFP–MreB protein fusion from the native locus (12) by epi-fluorescence microscopy (see “Materials and Methods”). MreB rotation depends on cell-wall insertion (33, 34), and the number of moving MreB filaments is linearly correlated with the rate of cell-envelope growth, if growth rate is modulated through nutrient quality (17). We, thus used MreB rotation as a readout for ongoing cell-wall insertion. More specifically, we measured the product of MreB-filament density (number of filaments per cell-contour area) times average speed, by simply summing up all MreB-track lengths and dividing by 2D cell area (contour area) and movie duration. We refer to this quantity as “MreB activity.” However, since diffraction-limited microscopy impedes the detection of all MreB filaments, we restricted our interpretation to large relative changes of MreB activity.

In agreement with previous observations (33,34), all drugs used here (vancomycin, penicillin G, D-cycloserine, and bacitracin) stop MreB motion within 4 to 8 min after adding the drug on top of the agarose pad (Fig. 3C and D; Figure S3D; Video S2). The delay is likely entirely due to the time it takes the drugs to diffuse to the cells. Since MreB rotation only depends on cell-wall insertion through the rod complex but not through class-A PBPs, we also treated the Δ4 strain lacking all class-A PBPs with vancomycin and observed qualitatively the same result as in wild-type cells (Figure S3E). Our experiments thus suggest that cell-wall insertion is either inhibited or drastically reduced at the time of MreB-motion arrest, while cell-wall expansion continues by about 10% to 20% during the residual time of observation (Fig. 3E).

We then wondered whether a surface-area increase observed after MreB-motion stop could be caused by an increase of turgor pressure alone, without any hydrolytic activity of cell-wall cleaving hydrolases. We, thus conducted hypo-osmotic shocks (Figure S3F–S3H) similar to (11). Those demonstrate that large increases in osmotic pressure (0.74 Osm) only lead to small increases in surface area (2% to 3%), which are about 5-fold smaller than after drug treatment (see also (11)). Thus, increasing surface area by 10% to 20% through turgor pressure would require the unphysiologically high accumulation of osmolytes. Furthermore, hypo-osmotic shocks lead to an increase of both cell length and cell width (Figure S3F and S3G), while width does not systematically change after drug treatment (Figure S3H). Thus, the observed increase of surface area by 10% to 20% after arrest of cell-wall insertion cannot be reconciled by an increase of turgor pressure alone, but instead requires that autolytic enzymes cut load-bearing cell-wall bonds. However, as the cell continues to grow, the peptidoglycan density is continuously reduced, which implies that less and less autolytic activity is required to expand the cell wall.

By comparing the time-dependent rates of surface growth and MreB activity at early times after different drug treatments, we also found that surface expansion proceeds at an unperturbed rate for about 2 to 6 min after MreB motion has stalled (Fig. 3D; Figure S3D), in qualitative agreement with previous observations (24) of the experimental data by (33). Thus, cell-surface growth and cell-wall insertion are not strictly coupled.

Next, we wondered whether we could find additional conditions under which cell-wall expansion and cell-wall insertion are decoupled. It was previously shown at the population-level that the inhibition of protein translation through chloramphenicol leads to a rapid reduction of biomass growth (based on turbidity), while peptidoglycan synthesis continues (35) and cell-wall thickness increases (36). Here, we investigated single cells treated with 100 *μ*g/ml of chloramphenicol, which completely inhibits protein translation (Figure S3I), in time-lapse microscopy. In agreement with (35), we observed that mass growth and cell-surface growth are strongly reduced (Fig. 3F), while MreB activity remains high (Fig. 3G; Video S3). Interestingly, cell-envelope and biomass growth remain coupled despite the severe perturbation. This coupling is also observed if we correct our calculation of cytoplasmic surface area and mass for cell-wall thickening (see “Materials and Methods”), a consequence of continued cell-wall insertion. This correction has only a small influence on the surface-to-mass ratio. Thus, our observations suggest that cells can regulate surface expansion through a pathway that is different from cell-wall insertion. Furthermore, our observation also demonstrates that the insertion of new envelope proteins is not required and, therefore, not rate-limiting for surface growth.

Together, the rates of cell-wall expansion and cell-wall insertion are not strictly coupled, suggesting that the activity of cell-wall-cleaving hydrolases is controlled by a pathway that is independent of cell-wall insertion.

### Inhibition of membrane synthesis also decouples surface expansion and mass growth

**Fig. 4.**
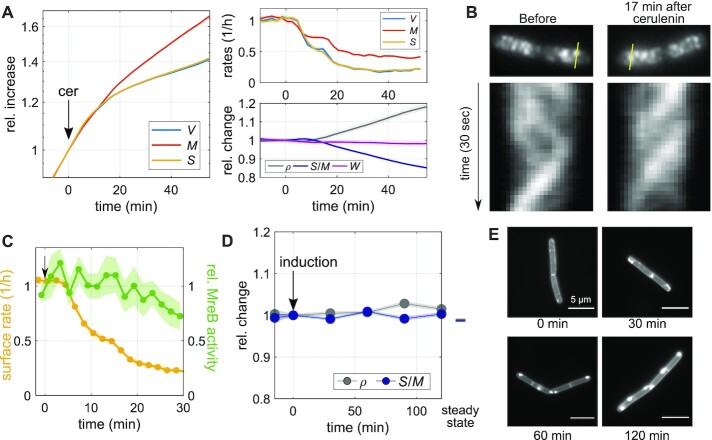
Inhibition of membrane synthesis decouples surface expansion and mass growth independently of peptidoglycan synthesis. (A) Single-cell time lapse of filamenting cells (bAB56) grown in S7_50_+GlcCaa and treated with cerulenin 100 *μ*g/ml (added to the agarose pad at time = 0). Relative increase (left) and rates (top right) of volume, surface area, and dry mass. Relative change (bottom right) of dry-mass density, surface-to-mass ratio, and width. (Solid lines + shadings = average ± 2*SE) (B) Kymographs of MreB-GFP rotation in filamenting cells (bYS19) during 30 s movie before and 17 min after cerulenin addition along the lines indicated in MreB-GFP snapshots (top). (C) Comparison of surface expansion rate (yellow; as in A) and relative MreB activity (green line + shading = average ± SE) of cerulenin-treated cells to that of nonperturbed cells, similar to Fig. 3(D). (D) Relative change of average dry-mass density and surface-to-mass ratio upon overexpression of AccDA (bSW305: *amyE*::pXyl-*accDA*) by addition of xylose (10 mM) at time = 0 in LB medium (average ± SE). Every point represents the average obtained from snapshots of batch-culture-grown cells. (E) AccDA overexpression in bSW305 (the same experiment in D) leads to the accumulation of excess membrane according to staining with the membrane dye MitoTracker green.

A different envelope component was recently demonstrated to have an important influence on cell-envelope growth (11): the cytoplasmic membrane. We, therefore, investigated how a modulation of membrane synthesis affects surface expansion. First, we treated cells with the fatty-acid-synthesis inhibitor, cerulenin (37), which quickly arrests lipid synthesis (Figure S4A). Similar to our experiments with cell-wall-synthesis inhibitors, we first added cerulenin at 100 *μ*g/ml to the top of an agarose pad, which then reaches the cells through diffusion on the timescale of few minutes. Within 7 min after drug addition, both surface expansion and mass growth are reduced (Fig. 4A; Video S4), qualitatively similar to the inhibition of peptidoglycan insertion (Fig. 3A). However, different from the inhibition of cell-wall insertion, cerulenin does not cause visible lysis or partial loss of mass and turgor. Since surface growth is affected more severely than mass growth, *S*/*M* decreases and mass density increases. We observed a very similar behavior when the drug was already contained in the agarose pad, that is, when the cells were immediately exposed to the drug at its final concentration (Figure S4B), apart from the initial diffusion-caused delay (Fig. 4A). Thus, membrane synthesis is required for the maintenance of *S*/*M*. However, surface growth continues at a reduced rate at least for 40 min, even though membrane synthesis is completely inhibited.

If cerulenin is washed out (after cerulenin-treatment for 30 min in bulk) *S*/*M* recovers within about one mass-doubling time (Figure S4C and S4D). During recovery, surface growth rate transiently exceeds mass growth by about 20% (Figure S4C), before returning to the rate of mass growth. These observations are consistent with the previous study by (23), which shows rapid resumption of membrane and biomass synthesis during refeeding of glycerol after transient glycerol starvation.

Given the similarity between cerulenin treatment and our inhibition of cell-wall synthesis (Fig. 3A), we wondered whether cerulenin might lead to a reduction of surface growth by affecting cell-wall insertion. We, thus monitored MreB-GFP activity as in Fig. 3(C) and (D). We found that MreB activity is not affected for at least 15 min after the initial reduction of surface expansion (Fig. 4B and C; Video S5), followed by a mild reduction during the remaining observation time. We confirmed these results with a complementary method developed by some of us (12). The method yields area density and speed of moving MreB filaments based on total-internal reflection microscopy (TIRF) and a subsequent kymograph-based analysis (Figure S4E). The method, which was previously shown to compare well with independent high-resolution structured-illumination microscopy (12), confirms our results.

Our observations, therefore, suggest that membrane synthesis affects surface expansion independently of peptidoglycan insertion. Our finding is in agreement with previous work from Mindich (23), who showed that cell-wall synthesis continues after membrane synthesis is inhibited by glycerol starvation, according to the incorporation of radioactive alanine (23).

The rate of mass growth slows down as time progresses. We initially speculated that this decrease might be a consequence of increased crowding. However, when inspecting the relationship between mass growth rate and mass density at the single-cell level, we observed no visible correlations (Figure S4F), supporting our conclusion drawn from the constant growth rate after modulation of aPBP levels (Fig. 1H): mass density is likely not rate-limiting for mass-growth rate.

Next, we studied a previously described mutant (bSW305, *amyE*::pXyl-*accDA*) that overproduces membrane lipids when grown in LB medium (38). If the cytoplasmic membrane was rate-limiting for surface growth, we would expect an increase of *S*/*M* upon *accDA* induction. However, we did not observe any change of *S*/*M* both during steady-state growth or during the first 2 h after *accDA* induction (Fig. 4D). At the same time, we observed apparent excess cytoplasmic membrane according to membrane staining with MitoTracker green (Fig. 4E) demonstrating increased membrane production in agreement with previous investigations by electron microscopy (38). Thus, while proper membrane physiology is apparently important for cell-envelope growth, independently of peptidoglycan insertion, excess membrane production does not lead to an increase of surface growth.

### The inhibition of multiple envelope-synthesis pathways is required to stop envelope growth

**Fig. 5.**
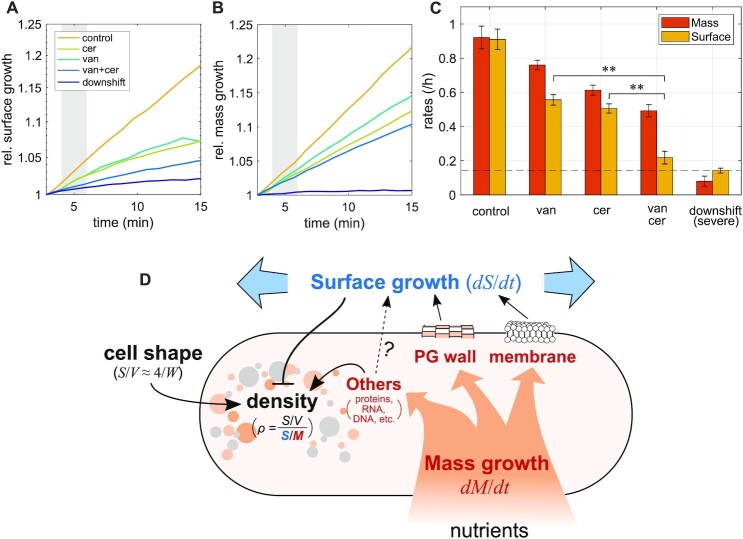
Simultaneous inhibition of multiple envelope-synthesis pathways. (A)–(C) Single-cell time lapse of wild-type cells on agarose pads (S7_50_+GlcCaa) that contain drugs (50 *μ*g/ml vancomycin; 100 *μ*g/ml cerulenin; and 50 *μ*g/ml vancomycin + 100 *μ*g/ml cerulenin) or alpha-MG for a severe nutrient downshift (see Fig. 2C). Relative increase of surface area (A) and dry mass (B). (Normalized by respective values at time = 3 ± 1 min) (C) Average rates of surface growth and mass growth calculated from the shaded regions in (A) and (B) (at about 5 min; error bars = ± SE). Dashed line indicates the surface-growth rate during severe nutrient downshift. The surface-growth rate during double drug treatment is significantly slower than those during single drug treatments (*P*< 0.01). (D) Model of the relationship between physiology, surface growth, and mass density in *B. subtilis*: nutrient uptake leads to mass growth, including macromolecules (proteins, RNA, DNA, and so on), peptidoglycan cell wall, and cytoplasmic membrane. Peptidoglycan insertion, membrane insertion, and possibly other processes (notably, envelope protein insertion) are required for surface growth. Because the surface-to-mass ratio is maintained approximately constant during perturbations of growth rate and cell shape, surface growth is thought to be controlled by mass growth. Cytoplasmic mass density is determined by cytoplasmic mass growth, surface growth, and cell shape according to a simple formula that relates density to the robust surface-to-mass ratio and to the surface-to-volume ratio.

From our perturbations of cell-wall and membrane insertion, we concluded that neither of these processes is solely rate-limiting for surface growth. We, thus wondered whether envelope expansion might respond additively to multiple envelope-insertion processes. To test this possibility, we inhibited cell-wall insertion and membrane insertion at the same time, by treating cells with vancomycin and cerulenin (Fig. 5A–C). The combined treatment indeed leads to a strong reduction of surface growth if compared to the single-drug treatments, within less than 3 min, approaching the surface growth rate observed after glucose starvation. This observation is compatible with the hypothesis that cell-wall insertion and membrane insertion account for most of cell-envelope growth in *B. subtilis* (Fig. 5D). How envelope expansion and envelope insertion are mechanistically coupled on the short timescales observed here remains to be discovered in future work.

## Discussion

In conclusion, cell-volume growth of the Gram-positive bacterium *B. subtilis* is indirectly determined by increasing surface area in proportion to biomass growth, qualitatively in the same way as the Gram-negative *E. coli* (9). More specifically, the surface-to-mass ratio *S*/*M* remains almost constant, independently of cell-to-cell variations of cell width or instantaneous growth rate. Since average width of *B. subtilis* does not systematically change in different nutrient conditions (39), surface-to-mass coupling can guarantee density homeostasis during growth. However, if width is modulated by varying expression of class-A PBPs, mass density changes (Fig. 1D).

The constancy of *S*/*M* requires both proper cell-wall insertion and membrane synthesis. If either of those processes is arrested, surface growth is reduced (Figs. 3 and 4). Once the inhibition of surface growth is relieved, cells rapidly recover their steady-state surface-to-mass ratio by growing faster in surface than in mass. Neither cell-wall insertion nor membrane synthesis are solely rate-limiting for surface growth. However, if both processes are inhibited together, surface growth arrests nearly completely (Fig. 5), in quantitative agreement with a starvation experiment (Fig. 2C).

The conserved surface-to-mass coupling across Gram-negative and Gram-positive bacteria is remarkable given their fundamentally different envelope architectures. A nearly constant surface-to-mass ratio during the cell cycle was recently also observed in fission yeast cells (40), similar to our previous observations in *E. coli* (9). Thus, surface-to-mass coupling might be widely conserved beyond bacteria.

We previously reasoned, based on our findings in *E. coli* (9), that the coupling of surface and mass might have a metabolic origin: constancy of *S*/*M* would come about if cells devoted a constant fraction of newly acquired mass to one or multiple envelope components whose production are rate-limiting for surface growth. Here, we found that both cell-wall insertion and membrane synthesis are required for the maintenance of *S*/*M* in *B. subtilis*, thus providing two links between metabolism and cell-envelope expansion.

We, thus propose a model summarized in Fig. 5(D), where nutrient uptake leads to buildup of cytoplasmic biomass and the cell envelope. Cell-envelope synthesis, comprising cell wall, membrane, and proteins, is required for surface growth, even if individual components are not solely rate-limiting. Cytoplasmic biomass, surface area, and cell width (more generally, the surface-to-volume ratio) determine the average intracellular density.

### The role of cell-envelope synthesis for surface growth

The dependency of *S*/*M* on cell-wall insertion is qualitatively different from *E. coli*, which expands surface area independently of cell-wall insertion (19). However, when perturbing cell-wall insertion in *B. subtilis*, we also observed significant deviations between cell-wall expansion and MreB-based cell-wall insertion: Most notably, inhibiting peptidoglycan insertion causes a reduction of MreB rotation only after a short but significant delay of 2 to 6 min (Fig. 3D; Figure S3D), and cell-wall expansion continues at a reduced rate even after MreB rotation is completely arrested. Furthermore, cells start to shrink and lose part of their mass or even lyse about 20 min after drug exposure. These findings suggest that autolytic enzymes, which are physically responsible for cell-wall growth during drug treatment, do not directly depend on cell-wall insertion but are controlled through an unknown signal. The signal could be found in the cell-wall structure itself, which, in turn, is affected by the lack of cell-wall insertion. Alternative or additional signals could also play a role in controlling hydrolase activity.

Inhibition of fatty-acid biosynthesis through cerulenin treatment leads to an equally rapid reduction of surface growth as the inhibition of cell-wall insertion (Fig. 4A). The cytoplasmic membrane is thus arguably equally important for the regulation of surface growth and thus for cell-volume regulation as the cell wall. Previously, it was demonstrated by different groups that membrane tension and membrane fluidity are important factors that modulate cell-wall insertion, which might affect surface growth (11,21, 22). Here, we found that the inhibition of fatty-acid synthesis reduces surface growth through a mechanism that is different from cell-wall insertion (Fig. 4A–C), in qualitative agreement with (23). However, while membrane insertion is apparently required for proper surface growth, visible overproduction of membrane upon overexpression of the Acetyl-CoA carboxylase components AccDA does not lead to an increase rate of surface growth (Fig. 4D and E), even if excess membrane likely contributes to more surface area after protoplast formation (38). This finding suggests that the flux of total membrane lipids is also not the sole rate-limiting envelope component, just like peptidoglycan insertion is not solely rate-limiting. However, it remains possible that the synthesis of specific lipids has a rate-limiting role for envelope growth. The role of the cytoplasmic membrane thus deserves further investigation in the future.

How membrane synthesis and cell-wall synthesis are linked to biomass growth is a question that remains fundamentally not understood in any bacterium. The first committed steps of fatty-acid and phospholipid synthesis are likely the major pathway elements for the control of membrane synthesis (41–45). However, the signals responsible for controlling their activities largely remain to be identified (43). Furthermore, while multiple gene-regulatory feedbacks for cell-wall metabolism have been identified (46), the question of how cell-wall metabolism and mass growth are robustly coupled remains open. Recent work from some of us (17) has identified PrkC as an important regulator of MreB-based cell-wall insertion. Sun and Garner (17) suggested that PrkC senses the availability of lipid II, the precursor of peptidoglycan synthesis, and thus regulates the number of moving MreB filaments. However, it remains to be investigated if and how lipid II levels are an important factor to coordinate cell-wall insertion and biomass growth.

### Transient variations of the surface-to-mass coupling

For the Gram-negative *E. coli* and *Caulobacter crescentus*, we recently proposed a new surface growth law that relates the rate of surface growth (d*S*/d*t*) to the rate of mass growth (d*M*/d*t*) according to the equation d*S*/d*t* = αd*M*/d*t* (9). Near-constancy of *S*/*M* in *B. subtilis* suggests that this growth law is also conserved in *B. subtilis*, despite the fundamentally different envelope architectures of Gram-negative and Gram-positive bacteria. However, in an alpha-MG-based nutrient downshift (Fig. 2B) we observed small but significant oscillations of the surface-growth rate around the mass growth rate, which corresponds to time-dependent variations of the coupling constant α and, which induce relative variations of *S*/*M* by about 2%. In *E. coli*, we could explain transient variations of *S*/*M* after nutrient downshifts by the elastic shrinkage of the cell envelope due to a reduction of turgor pressure, while plastic envelope expansion continues in proportion to mass growth. However, in *B. subtilis*, the observed variations are likely not due to elastic changes of the cell envelope: First, we observe a slight increase of *S*/*M* right after the nutrient downshifts, which would require an increase of pressure rather than the expected decrease. Second, when reducing cytoplasmic pressure through hyperosmotic shocks, surface area does indeed shrink as expected (11). However, this shrinkage always comes with a reduction of width of about half the relative amount, which we did not observe during the nutrient downshifts (Figure S2). The oscillations of *S*/*M* are, therefore, likely caused by variations of plastic surface growth rather than elastic changes of envelope stretching.

How do transient variations in surface growth then come about? According to the surface-growth model introduced above, asymptotic recovery of *S*/*M* is expected to occur on a timescale that is equal to the inverse of the instantaneous mass-growth rate [dlog (*M*)/d*t*]^−1^ ≈ 120 min. Instead, we observe an oscillation with a period of about 40 min, which is indicative of feedback, possibly due to the accumulation of cell-envelope material, as previously suggested to occur after a transient reduction of envelope growth due to hypoosmotic shock (11). A rapid non-oscillatory recovery of 〈*S*/*M*〉 is also observed after transient cerulenin treatment (Figure S4C). Whether an envelope-synthesis pathway or other processes are responsible for the oscillations in surface growth rate after nutrient shifts and for rapid recovery after cerulenin treatment remains to be investigated in further detail.

In summary of this part, the surface-growth law initially proposed for *E. coli* appears suitable to describe envelope growth in the evolutionary distant *B. subtilis*, apart from transient oscillations of surface growth, which lead to small variations of 〈*S*/*M*〉 of the order of 2% in our experiments (Fig. 2).

### Systematic deviations of *S*/*M* after changes of cell width

While *S*/*M* is independent of stochastic cell-to-cell variations of width (Fig. 1C), a systematic increase of average width by PonA overexpression leads to a reduction of the average value of 〈*S*/*M*〉, and a decrease of width through deletion of PonA or all class-A PBPs leads to an increase of 〈*S*/*M*〉 (Fig. 1D). Different mechanisms might be responsible for this correlation: First, the modified ratio of MreB-based and class-A PBP-based cell-wall insertion is known to affect cell-wall architecture (12, 47), which might then affect autolytic activity. Second, changes of cell width and dry-mass density likely affect mechanical envelope stresses, which might also affect autolytic activity (13). PonA expression or depletion is also known to affect the expression of different cell-wall-related proteins through the sigma factor σ^I^ (46). Finally, the change of 〈*S*/*M*〉 could also be the result of a yet unknown feedback between mass density and cell-wall expansion. Further work will be required to understand the effect of class-A PBP expression on surface growth.

### How is mass growth affected by perturbations of envelope growth or crowding?

Interestingly, inhibition of cell-wall insertion or fatty-acid synthesis does not only reduce the rate of surface growth but also affects biomass growth rate (Figs 3A and 4A). Mass-growth rate drops synchronously with the reduction of surface growth (at our time resolution of 2 min), even if the reduction is less pronounced. At this time point, mass density is not visibly affected. The reduction of mass growth rate is, thus not a response to increased crowding but likely triggered by an active signaling pathway or possibly caused by deficiencies in nutrient uptake as speculated by (48).

Previous work demonstrates that the stringent response is required for cell survival after cerulenin treatment in both *B. subtilis* (49) and *E. coli* (50), suggesting a potential role in reducing biomass growth in response to the arrest of membrane synthesis. However, on the generation timescale, the stringent response is not required for the reduction of biomass growth in *B. subtilis* (49). Thus, one or multiple different pathways must be responsible. Sun and Garner (17) proposed that levels of the peptidoglycan precursor lipid II affect both cell-wall insertion and mass growth through the kinase PrkC. Interestingly though, we found that the reduction of mass growth coincides with the reduction of surface growth and not with the time of MreB-rotation arrest. There are, thus likely additional links between surface growth and mass growth. In any case, surface-to-mass coupling appears to be bidirectional, with biomass growth affecting surface growth, but surface growth also affecting biomass growth, even if to a lesser extent.

At long times after the inhibition of cell-wall insertion or membrane biosynthesis, mass density increases due to the differences between mass and surface growth rates. We initially speculated that increased crowding might cause a decrease of mass growth rate observed during late times of drug treatment. However, to our surprise, we found that mass growth rate remained constant during steady-state exponential growth at different levels of PonA induction or class-A PBP deletion (Fig. 1H), which can cause similarly high levels of dry-mass density as vancomycin/cerulenin treatment. Furthermore, single-cell mass growth rate does not visibly correlate with mass density at different times after cerulenin treatment (Figure S4F). Mass density and crowding are considered important determinants of biomass growth rate, for example through their effect on the diffusion of tRNA complexes (51) or through a potential effect on the density of metabolites (7,8). However, constancy of growth rate despite strong differences in density suggests that crowding or density are not limiting factors for growth rate in *B. subtilis* in our growth conditions. Nevertheless, we expect that density and crowding become limiting at significantly higher levels.

## Materials and methods

### Growth conditions and sample preparation

Cell cultures were grown from a single colony in liquid media at 30°C in a shaking incubator. We used three different growth media: LB (Luria–Bertani Miller medium), S7_50_+Glc (minimal medium as described in (52), except that 0.4% glucose and 20 mM glutamate were used rather than 1% and 0.1%, respectively), and S7_50_+GlcCaa (S7_50_+Glc supplemented with 0.4% casamino acids). Before microscopy, we kept cultures in exponential phase for > 10 mass doublings at OD600 < 0.3 through back-dilution.

For single-cell snapshots or time-lapse movies we immobilized cells under a prewarmed agarose pad [1.5% UltraPure Agarose (16500-500, Invitrogen)]. Microscopy in a 30°C incubator was started within 3 min after cells were placed on the agarose pad.

For time-lapse movies, images were taken every 2 min if not specified. To avoid cell division, we started inducing *mciZ* from an IPTG- or xylose-inducible promoter by adding 1 mM IPTG or 30 mM xylose to the culture prior to microscopy, and we added the inducer at the same concentration in the agarose pad during microscopy (for the time of inducer addition, see Table S1). While MciZ inhibits the formation of new septa, about one-third of cells still contained noncomplete septa when placing cells on agarose pads, according to FM 4-64-based membrane staining. Thus, some of the cells analyzed are likely separated by a septum at the end of the time lapses, even if they are not visibly separated according to their contour. For simplicity we considered possibly chained cells as single cells.

To stain the cytoplasmic membrane, 1 *μ*g/ml of FM 4-64 Dye (ThermoFisher, T13320) was contained in agarose media. For perturbations, we added the following compounds to the top of the agarose pad: alpha-MG (0.4%), vancomycin (50 *μ*g/ml), D-cycloserine (10 mM), penicillin G (500 *μ*g/ml), bacitracin (500 *μ*g/ml), chloramphenicol (100 *μ*g/ml), and cerulenin (100 *μ*g/ml).

### Strain construction

All strains used in this study derive from the wildtype PY79. Strains, plasmids, DNA fragments, and oligonucleotides are all described in Table S2.

bAB56 (*mciZ*::spec-pHyperSpank-*mciZ*) was generated upon transformation of PY79 with a four-piece Gibson assembly reaction that contained the following amplified fragments: upstream of the *mciZ* gene; spectinomycin-resistance cassette loxP-spec-loxP; the *lacI* gene and the pHyperSpank promoter with an optimized ribosomal binding sequence; and the *mciZ* coding region and downstream sequence.

bKY42 (*ponA*::kan) was generated upon transformation of PY79 with genomic DNA from bMD586 (12).

bSW164 (*pbpD*::lox72, *pbpG*::lox72, *pbpF*::lox72, *ponA*::kan, *amyE*::spec-pSpac-*mciZ*) was generated upon successive rounds of transformation of PY79 with genomic DNA from strains bMK258 (*pbpD*::erm), bMK260 (*pbpG*::erm), and bMK270 (*pbpF*::erm), described below, and bSW99 (*amyE*::spec-pSpac-*mciZ*) (53) and bMD599 (*ponA*::kan) (12). After each transformation, the erythromycin-resistance cassette was removed with plasmid pDR244 (54). bMK258 (*pbpD*::erm), bMK260 (*pbpG*::erm), and bMK270 (*pbpF*::erm) were generated upon transformation of PY79 with a three-piece Gibson assembly reaction that contained the following amplified fragments: upstream of the respective gene to be deleted; erythromycin-resistance cassette loxP-erm-loxP; and downstream of the respective gene.

bSW305 (*amyE*::tet-pXyl-*accDA*) was generated upon transformation of PY79 with a five-piece Gibson assembly reaction that contained the following amplified fragments: upstream of the *amyE* gene; tetracyclin-resistance cassette loxP-tet-loxP; the xylR gene and the pXylA promoter with an optimized ribosomal binding site; the *accDA* coding region; and downstream of the *amyE* gene.

### Microscopy

Except for the TIRF-based MreB density measurements (Figure S4E), microscopy was carried out on a Nikon Ti-E inverted phase-contrast and epi-fluorescence microscope that is additionally equipped with a module for SLIM (26) as described in detail in (9). The microscope is equipped with a temperature chamber (Stage Top incubator, Okolab) set to 30°C, a Nikon Plan Apo 100x NA 1.45 Ph3 Objective, a solid-state light source (Spectra X, Lumencor Inc. Beaverton, OR), a multiband dichroic (69002bs, Chroma Technology Corp., Bellows Falls, VT), and with excitation (485/25, 560/32) and emission (535/50, 632/60) filters for GFP and FM 4-64 imaging, respectively. Epi-fluorescent images were acquired with a sCMOS camera (Orca Flash 4.0, Hamamatsu) with an effective pixel size of 65 nm, while phase-contrast and quantitative phase images were obtained with another CMOS camera (DCC3260M, Thorlabs) with an effective pixel size of 87 nm. For SLIM measurements we took six consecutive images with a phase delay of nπ/2, where *n*= [1, 2, 3, 4, 5, 6], with 200 ms exposure each. Out of these, we obtained three phase images (from images 1 to 4, 2 to 5, and 3 to 6, respectively), and took the average to obtain the final phase image. Including delays due to software, the acquisition of one final phase image took < 3 s. Micro-manager was used to control the microscope and acquire images within MATLAB.

For the TIRF-based investigation of MreB rotation shown in Figure S4E, which was carried out in Ethan Garner’s lab, microscopy was carried out on a Nikon Ti phase-contrast and TIRF microscope, equipped with temperature control, a Nikon 100X NA 1.45 objective, and a sCMOS camera (Orca Flash 4.0, Hamamatsu) with an effective pixel size of 65 nm. Nikon NI Elements was used to control the microscope.

### Measurement of cytoplasmic contour, dimensions, surface area, and volume

Cell dimensions were obtained from phase-contrast images acquired using the SLIM module, essentially as described previously (9). Specifically, we used the MATLAB-based tool Morphometrics (25) to determine cell contours. The image-formation process through the microscope, but also the contour-finding routines of Morphometrics can bias and distort the contour. We correct and calibrate for this based on epi-fluorescence images of cells stained with the fluorescent membrane stain FM 4-64. Since the calibartion is generally cell-shape dependent, we collected FM 4-64 images for wild-type cells and ponA-expressing mutant cells (*yhdG*::cat-pHyperSpank-*ponA*, *ponA*::kan) with different levels of inducer grown in S7_50_+GlcCaa medium. For FM 4-64 image acquisition, we focused on the middle of the cell based on phase-contrast microscopy through the epi-fluorescence port, which yields a sharper cell contour than the SLIM module. To correct these images for diffraction, we simulated membrane-stained cells as described (9) using the MATLAB based tool BlurLab (25) and using the point-spread function (PSF) of the microscope [based on 100 nm fluorescent beads (TetraSpeck, ThermoFisher)]. We applied the correction found in silico onto membrane contours obtained by Morphometrics to obtain the true (physical) contour of the periphery of the cytoplasm. In addition to the epi-fluorescence images, we obtained phase-contrast images of the same cells using the SLIM module. We then overlaid the measured contour of the phase-contrast cell with the corrected membrane contour obtained from the membrane dye and measured their respective offset as a function of cell width and as a function of the distance from the cell pole.

This correction was used to correct the contours of all cells measured with the SLIM module. Finally, given the calibrated contours of the cell, we used Morphometrics to apply a mesh-grid of 1 px (87 nm) step-size. This routine also gives the centerline of the cell, which is used to determine cell length. We then assume cylindrical symmetry around the centerline and infer cell surface and cell volume from the sum of the surfaces and volumes of truncated conical wedges with height and width given by the meshes.

For the confirmation of continued MreB activity after cerulenin treatment through TIRF microscopy (Figure S4E), we also segmented phase-contrast images using the Morphometrics tool. However, those data were not calibrated against FM4-64 images. This is not relevant for the calculation of instantaneous surface-growth rate or density of moving MreB filaments.

### Experimental quantification of cell-wall dry mass

Cultures of wildtype and bMD586 were grown to exponential phase in 1 l of S7_50_+GlcCaa medium. For the bMD586 culture, *ponA* expression was induced by 1 mM IPTG. Once OD600 reached 0.3, cells were harvested and washed with Milli-Q water. The suspension of the cells in Milli-Q water was evenly divided into two. The harvested cells from one suspension were subjected to vacuum drying overnight and the dry weight was measured. The other suspension was subjected to sonication to break cells (complete cell disruption was confirmed by microscopy), and the insoluble fraction containing cell wall and wall-associated proteins (55) was washed by Milli-Q water. We confirmed that protein mass in the insoluble fraction per total cell mass was about 0.2% based on the Bradford Assay (Bio-Rad Laboratories, Inc.), and, thus the insoluble fraction predominantly consists of cell wall. After vacuum drying overnight, the dry weight of the cell-wall fraction was measured. The cell-wall content (ζ) is calculated as dry weight of the cell-wall fraction per dry weight of total cell suspension.

### Calculation of cytoplasmic dry mass from quantitative-phase images

The cytoplasmic dry mass is calculated as

$$
\begin{equation*}
M = (1-\zeta )\left(\frac{\lambda }{2\mathrm{\pi }\gamma }\phi +\frac{V \left(n_\mathrm{medium}-n_\mathrm{H_{2}O}\right)}{\gamma } \right).
\end{equation*}
$$

Here, λ = 635 nm is the central wavelength of light, *n*_medium_ and $n_{\rm H_2O}$ are refractive index of the medium and water, respectively, and ζ ≈ 0.14 is the fraction of biomass occupied by the cell wall (obtained from bulk experiments; see previous section). For a correction of this value due to cell-wall thickening during treatment with either chloramphenicol (Fig. 3F and G) or cerulenin (Fig. 4A) see the subsequent section.

In case of experiments using an agarose pad, we added *n*_agarose_ ( = 0.0020) to *n*_medium_. We measured *n*_medium_ using a refractometer (Brix/RI-Chek, Reichert). γ is the refraction increment of the cell, estimated below, and ϕ is the integrated phase obtained from the phase image, detailed below. We defined cytoplasmic dry mass as all the dry mass other than the cell wall. This approach overestimates cytoplasmic dry mass by up to 5%, which is the estimated mass fraction of periplasmic proteins (56) and lipoteichoic acid (57). Since those mass fractions are only poorly known, we do not correct cytoplasmic mass for their presence. We also note that relative changes of mass, mass density, and surface-to-mass ratio, are not affected by this simplifying assumption.

To calculate the refraction increment, we considered the reported composition of dry mass (58) and reported values for refraction increments (59–62) (Table S3). Within the uncertainty of the refraction increments for individual cell constituents, the weighted average refraction increment is between 0.175 and 0.182 ml/g. To account for the higher illumination wavelength of 635 nm used in our experiments, we further decrease the refraction increment by 1% (63). Thus, we arrive at the average refraction increment of γ = 0.177 ml/g, which we used for all conversions.

ϕ is the integrated phase obtained from the phase image, after correction for attenuation by optical artifacts of the microscope, notably the halo effect, as described previously (9). In brief, the integrated phase is underestimated by about 2-fold, but the precise attenuation depends on cell geometry. To correct for this attenuation, we conducted computational simulations of phase images for every cell and every time point that are informed by the properties of the microscope and by the cytoplasmic contour. We then integrated the measured phase in simulated images and compared this value to the expected integrated phase from the simulation parameters (ground truth). This comparison yields an attenuation factor used to correct the underestimated integrated phase from experiments. We repeated this procedure for every cell and every time point.

Strictly speaking, the attenuation factor should be calculated based on the contour of the cell (rather than the contour of the cytoplasm). However, the attenuation factor changes by less than 1.5% if we assume a contour that is larger (in radial direction) by 56 nm, the sum of a potential periplasm (22 nm according to (64), but not observed in cryo-electron tomography by (65)) and cell wall (34 nm according to (66)). Due to the uncertainty about exact envelope geometry, we thus decided to ignore this effect in our calculations.

### Estimation of the effect of cell-wall thickening for the calculation of cytoplasmic mass and surface area

During chloramphenicol or cerulenin treatments (Figs 3F, G, and 4) cell-wall synthesis remains high according to MreB activity while surface-growth rate drops. Accordingly, cell-wall thickness is expected to increase. However, in our calculation of cytoplasmic mass *M*, we assume a constant mass fraction of the cell wall (14%). Furthermore, in our calculation of the cytoplasmic surface area we implicitly assume a constant distance between the cell contour and the cytoplasmic contour, since our cytoplasmic-contour estimate is based on phase-contrast images that are calibrated with the membrane stain FM4-64 in untreated cells.

To estimate the quantitative effect of these two errors on *S*/*M*, we consider a simple model for corrected cytoplasmic mass and cytoplasmic geometry during excess peptidoglycan synthesis. This then allows us to test and demonstrate that the coordinated increase of surface and mass during drug treatment remains valid, independently of the approximation. Furthermore, surface and mass remain coupled if we consider the ratio of cytoplasmic surface area and total mass *M*_tot_ = *M* + *M*_cellwall_ (instead of the ratio between cytoplasmic surface area and cytoplasmic mass).

We assume that the amount of cell-wall material per surface area and the thickness of the cell wall each increase in direct proportion to the difference between MreB activity *m* (defined in the “Materials and Methods” section “Measurement of MreB motion”) and surface growth $\lambda _S=(\rm {d}S/\rm {d}t)/S$ according to $\dot{\zeta }= (m(t)/m_0)\lambda _S^0-\zeta \lambda _S(t)$, where *m*_0_ and $\lambda _S^0$ are the unperturbed MreB activity and surface growth rate, respectively. Here, we made sure that $\dot{\zeta }=0$ during unperturbed growth. Furthermore, both cell-wall mass per total mass and cell-wall thickness are obtained in relative terms by setting ζ_0_ = 1. Thus, ζ is a time-dependent correction factor for both the cell-wall mass per total mass and for cell-wall thickness.

Due to the expected thickening of the cell wall, the contour of the cytoplasm is expected to be more distant from the cell contour, by the same absolute amount as the cell wall thickens. The same correction leads to a smaller cytoplasmic surface area *S*_corr_.

Applying the model for treatment with chloramphenicol, we found that relative changes of *S*_corr_/*M*_corr_ and *S*_corr_/*M*_tot_ do not deviate from that of *S*/*M* by more than 1.5%. Our studies of surface-to-mass coupling after drug treatment (Figs 3F, G, and 4) are, therefore, hardly affected by our approximation of cytoplasmic contour and mass.

### Immersive refractometry

For immersive refractometry, we immobilized cells in flow chambers (sticky-slide I Luer 0.1, Ibidi) with a 24 × 60 mm coverslip (Corning No 1.5) coated with APTES ((3-Aminopropyl)triethoxysilane, Sigma-Aldrich, A3648-100ML): The coverslips were incubated with 2% APTES in ethanol (v/v) for 15 min at RT; they were washed with ethanol three times and with distilled water once and then stored in ethanol; and before use, ethanol was dried with compressed air. After cell loading we exchanged the media with different refractive index adjusted by Ficoll 400 (Sigma-Aldrich, F4375-100G) and took phase-contrast images. The refractive index of the media including Ficoll 400 was measured with a refractometer (Brix/RI-Chek, Reichert). The focal plane was positioned at the middle of the cells.

### Growth analysis

For bulk growth analysis, cells were cultured in a test tube at 30°C and optical density at 600 nm (OD600) was recorded using a spectrophotometer (Eppendorf). To obtain doubling time, we fit an exponential function to the data points corresponding to the exponential phase (at OD600 between 0.03 and 0.3). For growth analysis from time-lapse microscopy, we calculated relative rates as d(log *X*)/d*t*(*t*_*i* + 0.5_) = 2(*X*_*i* + 1_ − *X*_*i*_)/(*X*_*i* + 1_ + *X*_*i*_)/Δ*t*_*i*_, where *X* = *V*, *S*, *M*, *t*_*i* + 0.5_ = 0.5(*t*_*i*_ + *t*_*i* + 1_), and Δ*t*_*i*_ = *t*_*i* + 1_ − *t*_*i*_. For the display of relative changes of *V*, *M*, *S* and other quantities, we linearly extrapolated single-cell quantities to *t* = 0 unless stated differently. For display, both relative changes and rates were smoothened with a Gaussian filter with standard deviation of 0.5, if not specified.

### Theoretical model of the relationship between *S*/*M* and cell-wall thickness upon *ponA* induction

To understand how cell-wall thickening might lead to the decrease of *S*/*M* we consider the following simple model: The mass of the cell wall *M*_cellwall_ can be described as *M*_cellwall_ = *Sh*ρ_cellwall_, where *h* is the cell-wall thickness and ρ_cellwall_ is the dry-mass density of cell wall. Solving this equation for *S*, we then obtain the surface-to-mass ratio as *S*/*M* = *M*_cellwall_/(*Mh*ρ_cellwall_). According to our measurements of the total amount of cell wall (Fig. 1F), the ratio of *M*_cellwall_/*M* is not affected by *ponA* induction. Assuming that dry-mass density of the cell wall ρ_cellwall_ does not change upon *ponA* induction, a decrease of *S*/*M* indeed corresponds to an increase of cell-wall thickness *h* as previously demonstrated (12). Note that the increase of cell-wall thickness alone cannot explain the increase of cell width after *ponA* induction by about 300 nm: with a relative decrease of *S*/*M* of about 20% upon *ponA* induction, cell-wall thickness is expected to increase by less than 10 nm [given previous reports of the cell-wall thickness of about 34 nm (66)], which is much smaller than the change in cell radius.

### NaCl-based osmotic shocks

The wild-type cells were immobilized in flow chambers as described above. We first grew cells in S7_50_+GlcCaa for hyper-osmotic shocks or in S7_50_+GlcCaa of 0.6 Osm or 1 Osm adjusted by NaCl for hypo-osmotic shocks, with a flow of 50 *μ*l/min. To perform osmotic shocks, we switched the medium to S7_50_+GlcCaa with different concentrations of NaCl with a flow of 500 *μ*l/min. We took time-lapse movies every 20 s.

### Measurement of MreB motion

We measured MreB motion in two different ways: Method (A) using an epi-fluorescence-based method described below and Method (B) a TIRF- and kymograph-based method reported previously (12). The former method is implemented on the same microscope used to conduct all quantitative-phase microscopy. The latter method is implemented on a microscope in Ethan Garner’s lab and was previously demonstrated to give results that agree with high-resolution structured-illumination microscopy (SIM-TIRF) (12). Except for the experiment shown in Figure S4E, we used Method (A). For epi-fluorescence-based quantification (Method A), we took epi-fluorescence images of MreB-GFP (every 1 s for 30 s) close to the bottom of the cells (about 250 nm below the central plane of cells). Additionally, we took a phase-contrast image to measure the cell contour using the Morphometrics package (25) as above. Peak detection and tracking of MreB-GFP were carried out by the Fiji plugin TrackMate (67). Fluorescence spots were detected using the Laplacian of Gaussians (LoG) detector, with a 0.3-*μ*m spot diameter. Tracks were generated using the linear motion LAP tracker, with a search radius 0.15 *μ*m, a minimum displacement of 0.2 *μ*m, and 1 frame gap allowed. To quantify the activity of MreB-based cell-wall insertion activity, we measure the sum of all MreB track lengths and divided by total segmented cell area and total observation time (30 s). We refer to this quantity as “MreB activity.” If we were able to track all MreB filaments in the field of view, this quantity would be proportional to the areal density of moving filaments times average speed.

For the TIRF-based quantification (Method B), we took images of MreB-GFP by TIRF microscopy (every 300 ms with 1 s interval for 2 min) followed by a phase-contrast image, and we analyzed the density of directionally moving MreB filaments during cerulenin treatment by the same methods reported by (12). In brief, for every position along the cell centerline, we created a kymograph and subsequently detected moving MreB filaments as described. We counted the directionally moving MreB filaments and then normalized by the projected cell area according to Morphometrics-based segmentation of phase-contrast images obtained at the end of the time-lapse movie to calculate the density of moving MreB filaments.

### Measurement of protein concentration

bAB56 cells were cultured in 50 ml of LB medium at 30°C. Chloramphenicol, 100 *μ*g/ml, was added to the culture at time = 0 min in Figure S3I. At each time point, cells were harvested from 2 ml of the culture by centrifugation and were suspended in 200 *μ*l of 6 M urea solution. After sonication of the suspension, protein concentration was measured using the Quick Start Bradford Protein Assay (Bio-Rad Laboratories, Inc.)

### Measurement of lipid synthesis during cerulenin treatment

Wild-type cells were cultured in S7_50_+GlcCaa. At time = 0, cells we added 2 *μ*Ci/ml of [^14^C]-acetate and cerulenin (or no cerulenin in the control; Figure S4A). We harvested cells at different time points as indicated and extracted total lipids using the method by Bligh and Dyer (68). Briefly, 0.8 ml of cell cultures were resuspended in 3 ml of chloroform-methanol (1/2, v/v) and incubated over night at 20°C. The samples were centrifuged for 20 min at 5,000 × *g* and supernatants were recovered and transferred to a new tube containing 1 ml of chloroform and 1 ml of 1M KCl. Samples were vortexed and centrifuged to separate the phases. The organic phase was then washed three times with 2 ml of 1 M KCl. The organic phase was evaporated under nitrogen dissolved in chloroform, and the radioactive content of the lipids was determined on a scintillation counter.

## Supplementary Material

pgac134_Supplemental_Files

## Data Availability

Single-cell data are freely available at https://doi.org/10.5061/dryad.6hdr7sr41.
