# Supplementary material for: The role of cell-envelope synthesis for envelope growth and cytoplasmic density in Bacillus subtilis
Source: PNAS Nexus. 2022 Jul 26;1(4):pgac134. doi: 10.1093/pnasnexus/pgac134 (PMC9437589; doi:10.1093/pnasnexus/pgac134)
Supplement: pgac134_Supplemental_Files [file pgac134_supplemental_files.zip › PNASNEXUS-PNASNEXUS-2022-00215-s01.pdf]

Supplementary Material

The role of cell-envelope synthesis for envelope growth and cytoplasmic density in *Bacillus subtilis*

Yuki Kitahara[1,2,3], Enno R. Oldewurtel[3], Sean Wilson[4,5], Yingjie Sun[4,5], Silvia Altabe[6], Diego de Mendoza[6], Ethan C. Garner[4,5], Sven van Teeffelen[1,3*]

Supplementary Figures

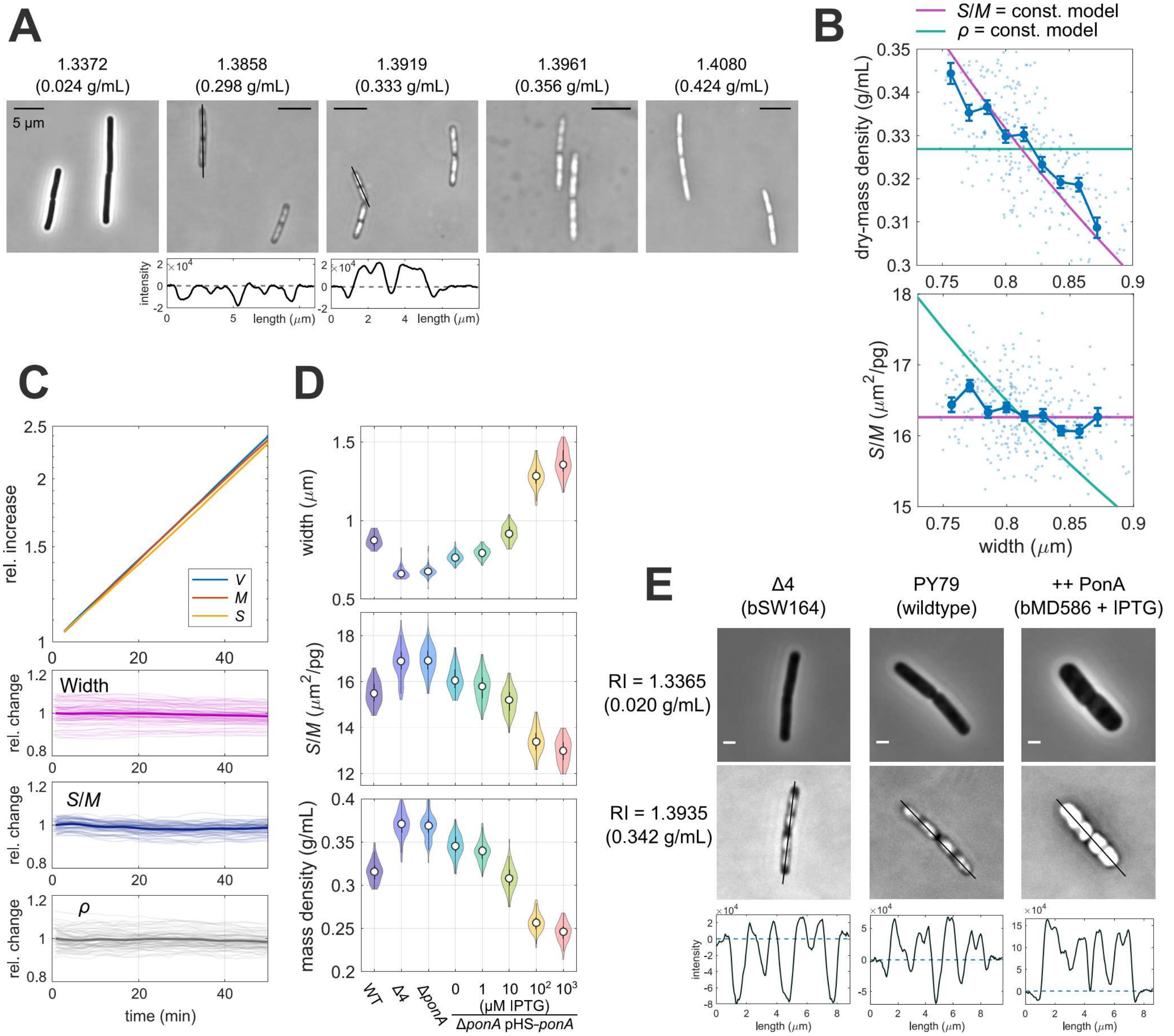

Figure S1. (Caption next page.)

Figure S1. Relationship between mass density, surface-to-mass ratio, and width in the wildtype and in aPBP mutants.

A: Confirmation of dry-mass density of cells during steady-state growth in LB medium by immersive refractometry. Top: Phase-contrast-microscopy snaphots of wild-type cells in flow chamber filled with different refractive indices (n =1.3372-1.4080) as indicated on top of images. Bottom: Intensity profiles along lines in the images demonstrate spatial heterogeneity of refractive index and thus mass density. If the average refractive index of cells is higher than that of the surrounding medium, the intensity inside cells is lower than outside (observed when n =1.3372 or 1.3858). If the refractive index of cells is lower than that of the surrounding medium, the intensity inside cells is higher than outside (observed when n =1.3919, 1.3961 and 1.4080). Thus, the average refractive indexof cells is in the range of 1.3858-1.3919, which corresponds to a mass-density range of 0.298-0.333 g/mL, compatible with Figure 1B.

B: Width dependency of dry-mass density and surface-to-mass ratio of wild-type cells during steady-state growth in $S7_{50}$+Glc. Blue dots: values of single wild-type cells; blue symbols and line: binned averages ± SE; pink lines: model prediction for spherocylinder with constant surface-to-mass ratio; green lines: model prediction for spherocylinder with constant dry-mass density.

C: Single-cell time lapse of filamentous cells (bAB56) on agarose pad ($S7_{50}$+GlcCaa). Top: Relative increase of volume, surface and dry mass (Solid lines + shadings = average ± 2*SE). Bottom: Relative change of single-cell width, surface-to-mass ratio, dry-mass density. All values are normalized with respect to the respective average values at time = 0 for better comparability.

D: Width (top), surface-to-mass ratio (mid), dry-mass density (bottom) under different expression levels of aPBPs (the same experiment shown in Figure 1G). Violin plots with median (white circles) and interquartile range (grey rectangles).

E: Immersive refractometry of wildtype and mutants with different aPBP-expression levels. Snapshots of wild-type, bSW164 and bMD586 cells after steady-state growth in $S7_{50}$+GlcCaa medium immobilized in flow chamber. To overexpress PonA, bMD586 cells was cultured with 1 mM IPTG. Top: Snapshots of cells in flow chamber filled with media of different refractive indices due to supplementation with Ficoll 400 (n =1.3365, 1.3935) taken by phase-contrast microscopy. Bottom: Intensity profiles along lines in top panels.

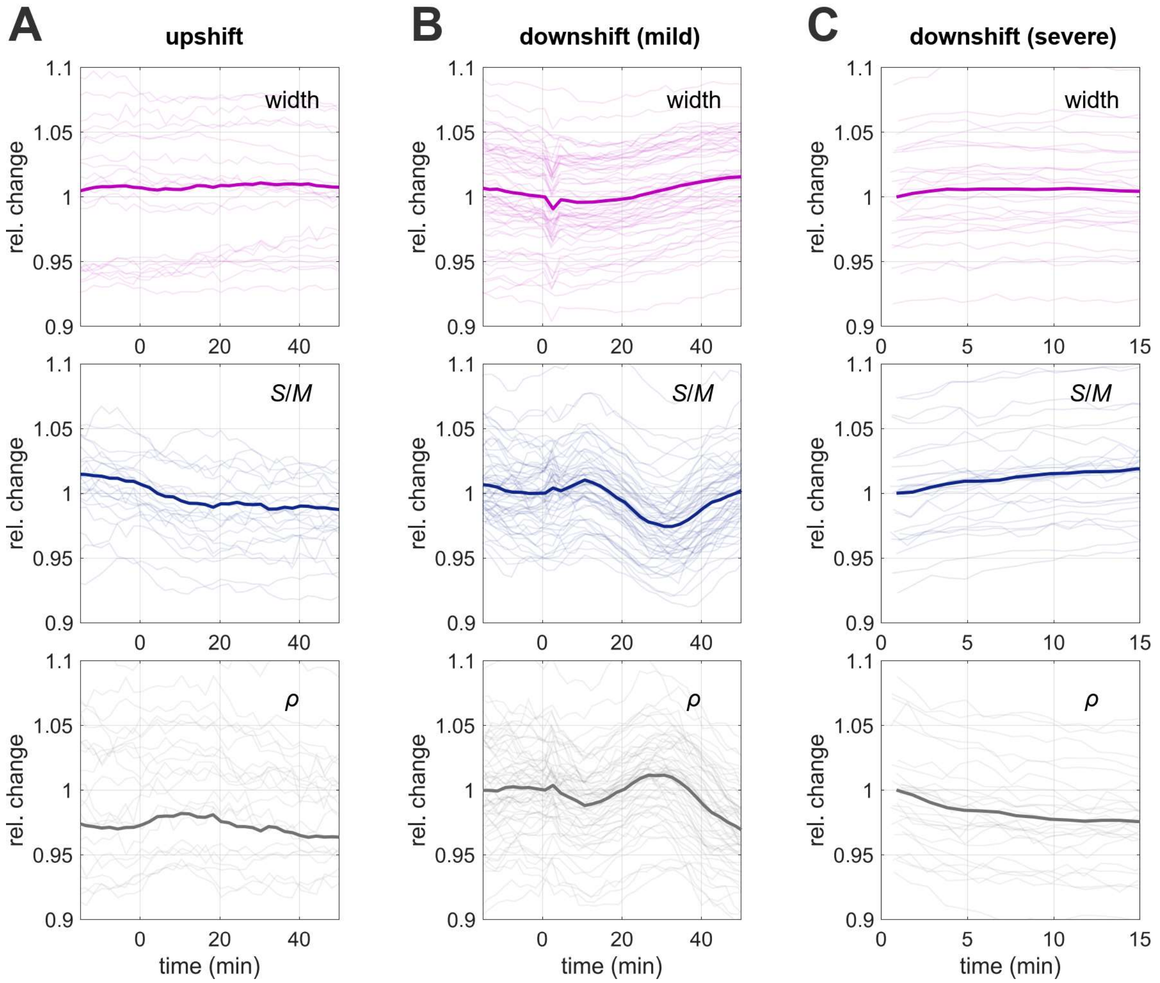

Figure S2. Single-cell traces during nutrient shifts.

Single-cell values of width (top), surface-to-mass ratio (middle), and dry-mass density (bottom) during nutrient upshift (the same experiment shown in Figure 2A) (**A**), during mild nutrient downshift (the same experiment shown in Figure 2B) (**B**), and during severe nutrient downshift (the same experiment shown in Figure 2C) (**C**). All values are normalized with respect to the respective average values at time = 0 for better comparability.

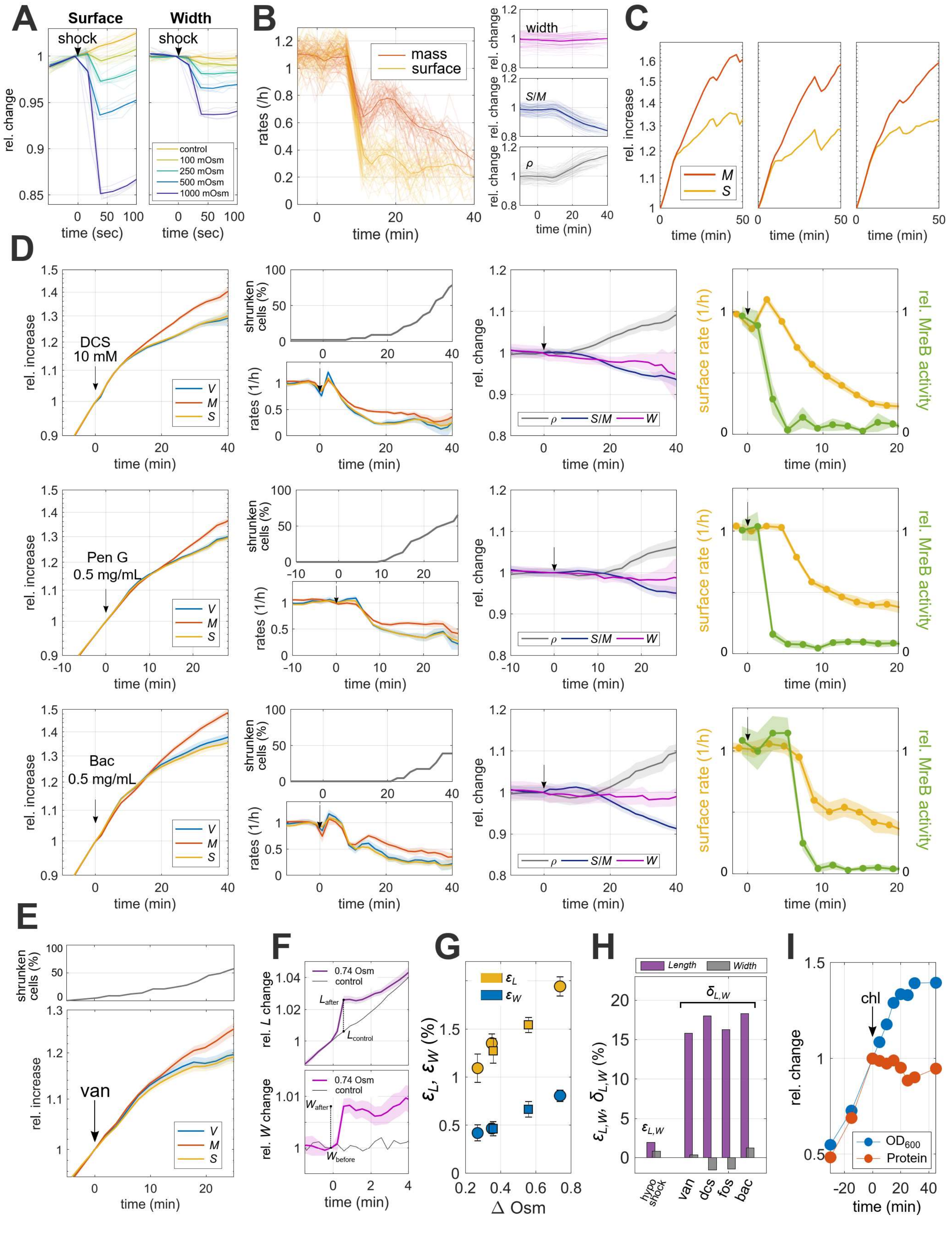

Figure S3. (Caption next page.)

Figure S3. The roles of pressure, cell-wall synthesis, and protein synthesis for surface growth.

A: Relative changes of surface area and width after NaCl-based hyper-osmotic shocks of wild-type cells in flow chamber (media changed from $S7_{50}$+GlcCaa medium to $S7_{50}$+GlcCaa supplemented with different NaCl concentrations at time = 0) demonstrate that a shock of 100 mOsm or more leads to a visible reduction of width. Thin lines: single cells. Bold lines: averages over single cells.

B-C: Single-cell behavior during vancomycin treatment (the same experiment of Figure 3A, B). **B:** Left: Relative rates of mass and surface area. Right: Relative changes of width, surface-to-mass ratio, and dry-mass density with respect to the respective average values at time = 0. Thin lines: single cells. Bold lines: averages over single cells.**C:** Relative increase of surface and dry mass of single cells demonstrate a transient reduction of dry mass and osmotic pressure.

D: Single-cell time lapses and MreB activity during inhibition of peptidoglycan synthesis using D-cycloserine (10 mM) (top), Penicillin G (0.5 mg/mL) (middle) and Bacitracin (0.5 mg/mL) (bottom). Columns 1-3: Single-cell time lapses of filamenting bAB56 cells treated with different drugs in analogy to vancomycin treatment shown in Figure 3A, B. Relative increase (1st column) and rates (2nd column-bottom) of volume, surface and dry mass. Lysis rates (2nd column-top). Relative change of dry-mass density, surface-to-mass ratio, and width (3rd column). (Solid lines + shadings = average ± 2*SE) Column 4: MreB activity activity (green line + shading = average ± 2*SE) , right axis) obtained from MreB-GFP movies and surface-expansion rate (yellow, left axis) of bYS19 cells obtained as in Figure 3D

E: Single-cell time lapse of Δ4 strain during vancomycin treatment (50 μg/mL). Relative increase (top) of volume, surface and dry mass and lysis rates (bottom). (Solid lines + shadings = average ± 2*SE)

F-H: Hypo-osmotic shocks demonstrate that elastic cell-wall stretching cannot account for surface-area increase observed after inhibition of cell-wall insertion.

F: Relative length change (top) and relative width change (bottom) after 0.74 Osm-shock of wild-type cells growing in $S7_{50}$+GlcCaa supplemented with NaCl (1 Osm) in flow chamber (media changed to $S7_{50}$+GlcCaa) (Solid lines + shadings = average ± 2*SE). **G:** Relative increase of cell length ϵ_L and cell width ϵ_W after different NaCl-based hypo-osmotic shocks (squares: shocks from 0.6 Osm; circles: shocks from 1 Osm). Here, we defined $\epsilon_L = (L_{\text{after}} - L_{\text{control}})/L_{\text{control}}$ and $\epsilon_W = (W_{\text{after}} - W_{\text{before}})/W_{\text{before}}$ with L_{after}, L_{control}, W_{after}, W_{before} shown in F. This demonstrates that an instantaneous turgor increase stretches both cell length and cell width corresponding to an increase of surface area by about $\epsilon_L + \epsilon_W$ = 1.5-3% (depending on shock magnitude) and resulting in a ratio of $\epsilon_W/\epsilon_L \approx 2.5$ (independent of shock magnitude). (symbols and error bars: average ± SE) **H:** Relative increase of length and width after large hypo-osmotic shock (0.74 Osm) compared to relative increase of length and width after inhibition of cell-wall insertion. Dimension changes after drug treatments differ in terms of absolute magnitude and even sign from those observed after changes of turgor pressure (G), demonstrating that elongation during cell-wall synthesis arrest cannot be explained by turgor pressure alone. We defined relative changes of length and width after arrest of cell-wall insertion as $\delta_L = (L_t - L_0)/L_0$ and $\delta_W = (W_t - W_0)/W_0$, where L_0 and W_0 are measured at the time right before MreB arrest and t is the time where the largest cell length was observed.

I: Relative increase of optical density (OD600) and protein amount according to Quick Start Bradford assay during chloramphenicol treatment. Wild-type cells were cultured in LB medium, and 100 μg/mL of chloramphenicol was added at time = 0.

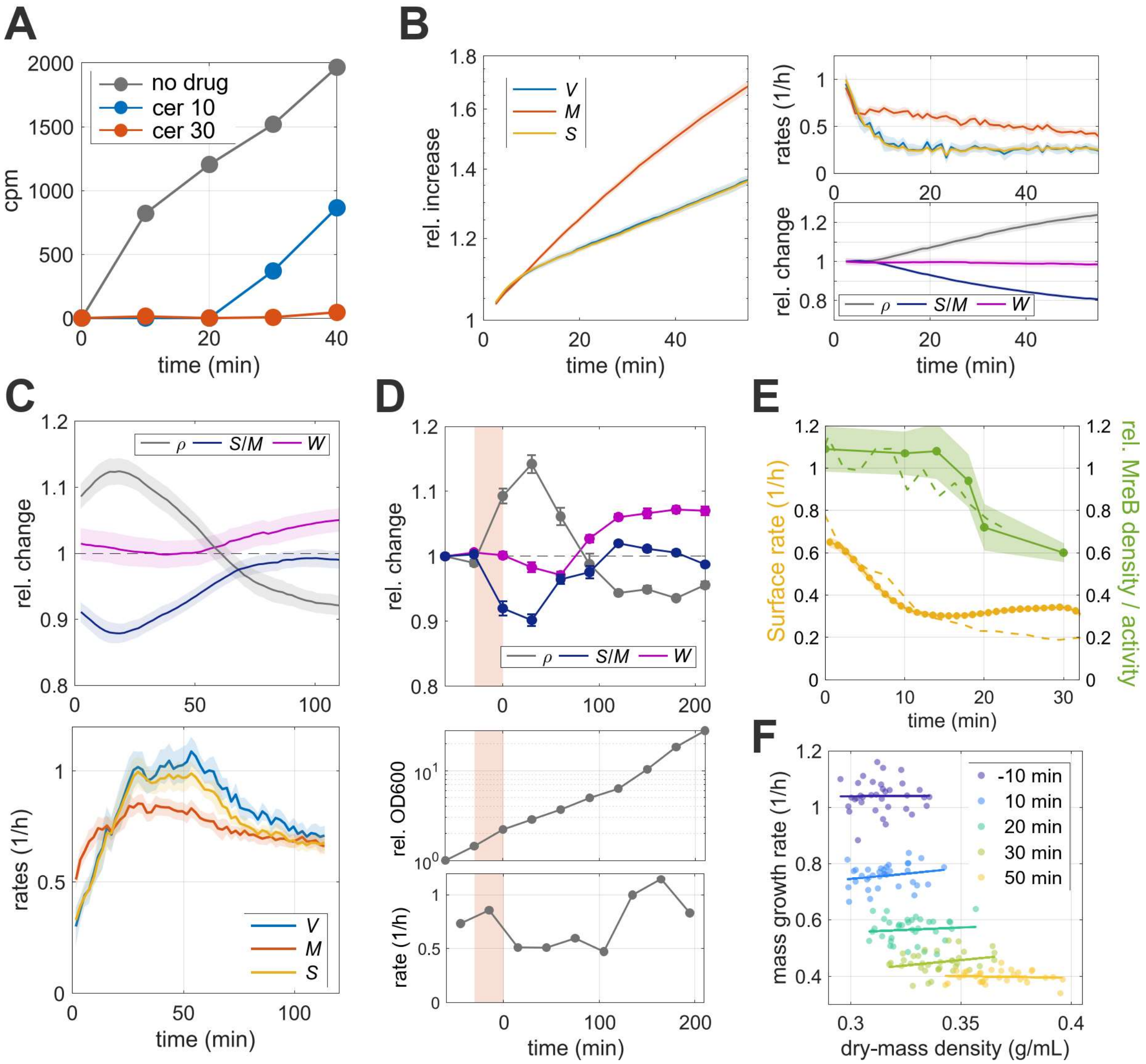

Figure S4. (Caption next page.)

Figure S4. Effect of cerulenin on surface growth.

A: Measurement of lipid synthesis by [^{14}C]-acetate incorporation after cerulenin treatment (0, 10, 30 μg/mL) during growth in $S7_{50}$+GlcCaa medium. Both [^{14}C]-acetate and cerulenin were added at time = 0. **B:** Relative increase (left) and rates (right-top) of volume, surface and dry mass, as well as relative change of dry-mass density, surface-to-mass ratio, and width (right-bottom) from single-cell time lapse during treatment with cerulenin (100 μg/mL) contained in the agarose pad (growth medium: $S7_{50}$+GlcCaa) prior to microscopy, such that cells are immediately exposed to the drug at its final concentration. (Solid lines + shadings = average ± 2*SE) **C:** Single-cell time lapse of bAB56 cells during recovery from 30 min cerulenin treatment (100 μg/ml) on an agarose pad in $S7_{50}$+GlcCaa. (Solid lines + shadings = average ± 2*SE) Analogous to panels in A.

D: The same recovery experiment as in B in batch culture, followed before, during (shaded region), and after cerulenin treatment. The culture was back diluted to keep optical density < 0.3. Left: Relative changes of average dry-mass density, surface-to-mass ratio, and width (average ± SE) obtained from single-cell snapshots (time = 0 corresponds to the time of washout). Right: Relative change of optical density (OD600), after normalization for backdilution, and growth rate.

E: Measurement of MreB-based cell-wall insertion after cerulenin treatment by complementary method. Solid lines: Surface expansion rate (yellow) and relative density of directionally moving MreB filaments (green) of bYS19 cells treated with cerulenin 100 μg/mL. Different from Figure 4, cerulenin was contained in the agarose pad ($S7_{50}$+GlcCaa). Density of directionally moving MreB filaments was measured based on TIRF-imaging and the analysis of kymographs [1]. Surface expansion rate was calculated based on time-lapse movies with 1 min interval. Dashed lines: For comparison, we also indicated surface expansion rate and relative MreB activity based on epi-fluorescence movies (see also Figure 4), with the time shifted by 7 min as an estimated time when cells are exposed to the minimal inhibitory concentration [2] according to a 1-dimensional diffusion equation. (Solid lines + shadings = average ± SE)

F: Relationship between dry-mass density and mass growth rate during cerulenin treatment. (the same experiment shown in Figure 4A). Dots: single-cell measurements, lines: linear regression. Mass growth rates were smoothened with a Gauss filter with standard deviation = 4 min.

Supplementary tables

Table S1. Detailed information of snapshot-, time-lapse-, and MreB-experiments.

Figure	Experiment	Strain	Medium	width * (μm)	S/M * (μm^2/pg)	ρ * (g/mL)	MreB activity or density *	t_{exp} ** (min)	Number of considered cells	t_{MciZ} *** (min)	biological replicates
1B, 1C	snapshot, steady state in S750 + GlcCaa	PY79	S750 + GlcCaa						217		†
1B, S1B	snapshot, steady state in S750 + Glc	PY79	S750 + Glc						315		†
1B	snapshot, steady state in LB	PY79	LB miller						29		†
S1C	timelapse, steady state in S750 + GlcCaa	bAB56	S750 + GlcCaa, 1mM IPTG	0.884	16.34	0.299		0	63	-30	†
1D	timelapse, *ponA* induction	bMD834	S750 + GlcCaa + 1mM IPTG, 30 mM xylose	0.755	17.022	0.331		0	34	-30	1E
1E	snapshot during *ponA* induction	bMD586	S750 + GlcCaa + 1mM IPTG	0.749	16.274	0.346			N = 38, 48, 29, 45, 46, 46, 34, 30, 24, 54<br>when t = -30, 0, 30, 60, 90, 120, 150, 180, 210, 240 min		1D
1G, 1H, S1D	snapshot, aPBP titration (WT)	PY79	S750 + GlcCaa						69		†
1G, 1H, S1D	snapshot, aPBP titration (0 mM)	bMD586	S750 + GlcCaa						94		†
1G, 1H, S1D	snapshot, aPBP titration (0.001mM)	bMD586	S750 + GlcCaa + 0.001 mM IPTG						77		†
1G, 1H, S1D	snapshot, aPBP titration (0.01mM)	bMD586	S750 + GlcCaa + 0.01 mM IPTG						82		†
1G, 1H, S1D	snapshot, aPBP titration (0.1mM)	bMD586	S750 + GlcCaa + 0.1 mM IPTG						89		†
1G, 1H, S1D	snapshot, aPBP titration (1 mM)	bMD586	S750 + GlcCaa + 1 mM IPTG						65		†
1G, 1H, S1D	snapshot, aPBP titration (Δ4)	bSW164	S750 + GlcCaa						65		†
1G, 1H, S1D	snapshot, aPBP titration (Δ*ponA*)	bKY42	S750 + GlcCaa						75		†
2A, S2A	timelapse, nutrient upshift	bAB56	S750 + Glc, IPTG 1mM (+ 0.4% Caa for upshift)	0.848	16.439	0.313		-30	22	-80	†
2B, S2B	timelapse, nutrient downshift (mild)	bAB56	S750 + GlcCaa, IPTG 1mM (+ 0.4% αMG for downshift)	0.851	15.665	0.313		-30	59	-60	†
2C, S2C, 5A-C	timelapse, nutrient downshift (severe)	PY79	S750 + 2% αMG	0.837	16.499	0.31		0	26		†
S3A	timelapse, hyper-osmotic shock (control)	PY79	S750 + GlcCaa						12		†
S3A	timelapse, hyper-osmotic shock (100 mOsm)	PY79	S750 + GlcCaa + 50 mM NaCl						14		†
S3A	timelapse, hyper-osmotic shock (250 mOsm)	PY79	S750 + GlcCaa + 125 mM NaCl						9		†
S3A	timelapse, hyper-osmotic shock (500 mOsm)	PY79	S750 + GlcCaa + 250 mM NaCl						6		†
S3A	timelapse, hyper-osmotic shock (1 Osm)	PY79	S750 + GlcCaa + 500 mM NaCl						6		†
3A-B, 3D-E, S3B-C	timelapse, vancomycin treatment	bAB56	S750 + GlcCaa, IPTG 1mM (+ 50 μg/mL vancomycin)	0.86	15.559	0.313		-30	63	-60	†
3C-E	MreB, vancomycin treatment	bYS19	S750 + GlcCaa (+ 50 μg/mL vancomycin)				1.325 μm^{-1}		N =16-11-10-12-6-8-8-6-7-10-8<br>every 2 min from t = -1~19 min		†
3E, S3D	timelapse, DCS treatment	bAB56	S750 + GlcCaa, IPTG 1mM (+ 10 mM D-cycloserine)	0.891	15.92	0.296		-30	44	-60	†
3E, S3D	timelapse, penicillin treatment	bAB56	S750 + GlcCaa, IPTG 1mM (+ 0.5 mg/mL penicillin G)	0.87	15.688	0.307		-30	46	-60	†
3E, S3D	timelapse, bacitracin treatment	bAB56	S750 + GlcCaa, IPTG 1mM (+ 0.5 mg/mL bacitracin)	0.872	16.077	0.3		-30	23	-60	†
3E, S3D	MreB, DCS treatment	bYS19	S750 + GlcCaa (+ 10 mM/mL D-cycloserine)				1.325 μm^{-1}		N =13-11-8-4-5-6-4-9-9-4-12<br>every 2 min from t = -1~19 min		†
3E, S3D	MreB, bacitracin treatment	bYS19	S750 + GlcCaa (+ 0.5 mg/mL bacitracin)				1.325 μm^{-1}		N =10-6-11-11-11-9-7-8-10-10-12<br>every 2 min from t = -1~19 min		†
3E, S3D	MreB, penicillinG treatment	bYS19	S750 + GlcCaa (+ 0.5 mg/mL penicillin G)				1.325 μm^{-1}		N =18-11-12-12-16-14-17-18-27-10-24<br>every 2 min from t = -1~19 min		†
S3E	timelapse, vancomycin treatment	bSW164	S750 + GlcCaa, IPTG 1mM (+ 50 μg/mL vancomycin)					-20	32	-60	†
S3F-H	timelapse, hypo-osmotic shock (from 0.6 Osm)	PY79	S750 + GlcCaa (0.6 Osm) → S750 + GlcCaa (0.6, 0.24, 0.04 Osm)<br>adjusted by NaCl addition or by water dilution						N = 16, 21, 22		†
S3F-H	timelapse, hypo-osmotic shock (from 1 Osm)	PY79	S750 + GlcCaa (1 Osm) → S750 + GlcCaa (1, 0.73, 0.65, 0.26 Osm)<br>adjusted by NaCl addition						N = 18, 12, 21, 23		†
3F, 3G	timelapse, chloramphenicol treatment	bAB56	S750 + GlcCaa, IPTG 1mM (+ 100 μg/mL chloramphenicol)	0.837	15.638	0.318		-30	24	-60	†
3G	MreB, chloramphenicol treatment	bYS19	S750 + GlcCaa (+ 100 μg/mL chloramphenicol)				1.325 μm^{-1}		N =9-10-15-9-12-6-10-8-15-7-8-10-7-6-11-13<br>every 2 min during t = -1~29 min		†
4A, 4C	timelapse, cerulenin treatment	bAB56	S750 + GlcCaa, IPTG 1mM (+ 100 μg/mL cerulenin)	0.868	15.285	0.315		-30	36	-60	S4E, S4B
S4B	timelapse, cerulenin treatment	bAB56	S750 + GlcCaa, IPTG 1mM containing 100 μg/mL cerulenin	0.876	15.687	0.312		0	29	-30	4A, S4E
S4C	cerulenin recovery	bAB56	S750 + GlcCaa, IPTG 1mM	0.803	16.554	0.319		0	23	-50	S4D
S4D	cerulenin recovery	PY79	S750 + GlcCaa	0.807	16.116	0.325			N =60-34-38-43-30-24-44-30-49-32<br>when t = -60, -30, 0, 30, 60, 90, 120, 150, 180, 210 min		S4C
4B, 4C	MreB, cerulenin treatment	bYS19	S750 + GlcCaa (+ 100 μg/mL cerulenin)				1.325 μm^{-1}		N =6-5-12-7-10-10-9-6-4-5-6-7-10-12-6-9<br>every 2 min from t = -1~29 min		S4E
S4E	timelapse, cerulenin treatment<br>(performed at Garner lab)	bYS19	S750 + GlcCaa containing 100 μg/mL cerulenin						38		4A, S4B
S4E	MreB, cerulenin treatment<br>(TIRF-imaging, performed at Garner lab)	bYS19	S750 + GlcCaa containing 100 μg/mL cerulenin				1.7 μm^{-2}		N =23-31-17-22-16-49<br>when t = 0, 10, 14, 18, 20, 30 min		4B
4D, 4E	snapshot during accDA overexpression	bSW305	LB miller + 10 mM xylose	0.845	15.781	0.307			N =10-38-41-55-29-33<br>when t = -15, 0, 30, 60 90, 120 min		†
5A-C	timelapse, control	PY79	S750 + GlcCaa					0	10		†
5A-C	timelapse, vancomycin treatment	PY79	S750 + GlcCaa containing 50 μg/mL vancomycin					0	26		†
5A-C	timelapse, cerulenin treatment	PY79	S750 + GlcCaa containing 100 μg/mL cerulenin					0	18		†
5A-C	timelapse, vancomycin and cerulenin treatment	PY79	S750 + GlcCaa containing<br>+50 μg/mL vancomycin +100 μg/mL cerulenin					0	19		†

* used for normalization
** time of placing cells on agarose pad
*** time of MciZ induction prior to placing cells on agarose pad
biological replicates:
†: confirmed by repeating the same experiment from independent cultures starting from separate colonies or by conducting multiple independent experiments with slight variations (e.g. different osmotic shocks Fig. S3)
Figure numbers: confirmed by very similar experiments as indicated.

Table S2. Oligonucleotides, DNA fragments and strains used in this study.

Category	Name	Sequence/Description/Genotype
Oligonucleotides	oAB49	GCTGTTTCCCCGCTTACAGC
Oligonucleotide	oAB50	GCTATACGAACGGTAGTTGACCAGTGCTCCCTGGAAGAAGCCCCCTTTTGCTC
Oligonucleotide	oSW40	CAGGGAGCACTGGTC
Oligonucleotide	oSW42	TTCTGCTCCCTCGC
Oligonucleotide	oMD232	GGTAGTTCCTCCTTAAAGCTTAATTGTTATCCGCTCACAAT
Oligonucleotide	oMD234	ATACGAACGGTACTGAGCGAGGGAGCAGAATAATGGATTTCCTTACGCGAAATACG
Oligonucleotide	oAB51	GCGGATAACAATTAAGCTTTAAGGAGGAACTACCGTGAAAGTGCACCGCATGCC
Oligonucleotide	0AB52	AACAAAAATCCATTGACAAACACCA
Oligonucleotide	oMK68	CCTCAGCATTTTCTTCATGGGCTTTG
Oligonucleotide	oMK179	ATACGAACGGTAGTTGACCAGTGCTCCCTGTCTCTTTTCTCCTAAATTCTAGCCATATC
Oligonucleotide	oJM28	TTCTGCTCCCTCGCTCAG
Oligonucleotide	oJM29	CAGGGAGCACTGGTCAAC
Oligonucleotide	oMK180	ATACGAACGGTACTGAGCGAGGGAGCAGAAAGGATCGGAGGGGATATGGAG
Oligonucleotide	oMK181	GCAAATACCGTGACCGAGCTG
Oligonucleotide	oMK173	ATACGAACGGTAGTTGACCAGTGCTCCCTGAACGGGTTCCCCCTTTTTTTATGTTTC
Oligonucleotide	oMK174	GCTTCATCAAATCTCATCTATAATACCC
Oligonucleotide	oMK175	ATACGAACGGTACTGAGCGAGGGAGCAGAAAAATAACCCGGCTCCTCGGAG
Oligonucleotide	oMK176	CGGAGAGCTACGGCTTTATCG
Oligonucleotide	oMK167	ATACGAACGGTAGTTGACCAGTGCTCCCTGGAACTCACCTCGCCTTTCTAAAG
Oligonucleotide	oMK168	CCTTCCGCTTACAGATTATTCATAG
Oligonucleotide	oMK170	ATACGAACGGTACTGAGCGAGGGAGCAGAATGGAATTCGGCGATTTTTTGAACTTTG
Oligonucleotide	oMK171	CAGGTTGCCCTGCACTGTTTTAG
Oligonucleotide	oMD191	TTTGGATGGATTCAGCCCGATTG
Oligonucleotide	oMD108	ACGAACGGTAGTTGACCAGTGCTCCCTGTCTTGACACTCCTTATTTGATTTTTTGAAGAC
Oligonucleotide	oSW38	CATTATACGAACGGTACTGAGCGAGGGAGCAGAAGAATTCGAGCTTGCATG
Oligonucleotide	oSW39	GGTAGTTCCTCCTTAATCG
Oligonucleotide	oSKH071	TTTGAATGGATCGATTAAGGAGGAACTACCTTGTTAAAGGATATATTCACG
Oligonucleotide	oSKH072	TCTTTCGGTAAGTCCCGTCTAGCCTTGCCCTTAGTTTACCCCGATATATT
Oligonucleotide	oMD196	GGGCAAGGCTAGACGGG
Oligonucleotide	oMD197	TCACATACTCGTTTCCAAACGGATC
DNA fragment	upstream of the *mciZ* gene	amplified from PY79 genomic DNA using primers oAB49 and oAB50
DNA fragment	spectinomycin-resistance cassette loxP-spec-loxP	amplified from pWX466 using primers oSW40 and oSW42
DNA fragment	*lacI* gene and pHyperSpank promoter with an optimized RBS	amplified from pDR111 using primers oMD234 and oMD232
DNA fragment	*mciZ* coding region and downstream sequence	amplified from PY79 genomic DNA using primers oAB51 and oAB52
DNA fragment	upstream of the *pbpD* gene	amplified from PY79 genomic DNA using primers oMK68 and oMK179
DNA fragment	erythromycin-resistance cassette loxP-erm-loxP	amplified from pWX469 using primers oJM28 and oJM29
DNA fragment	downstream of the *pbpD* gene	amplified from PY79 genomic DNA using primers oMK180 and oMK181
DNA fragment	upstream of the *pbpG* gene	amplified from PY79 genomic DNA using primers oMK174 and oMK173
DNA fragment	downstream of the *pbpG* gene	amplified from PY79 genomic DNA using primers oMK175 and oMK176
DNA fragment	upstream of the *pbpF* gene	amplified from PY79 genomic DNA using primers oMK168 and oMK167
DNA fragment	downstream of the *pbpF* gene	amplified from PY79 genomic DNA using primers oMK170 and oMK171
DNA fragment	upstream of the *amyE* gene	amplified from PY79 genomic DNA using primers oMD191 and oMD108
DNA fragment	tetracyclin-resistance cassette loxP-tet-loxP	amplified from pWX470 using primers oSW40 and oSW42
DNA fragment	*xylR* gene and the pXylA promoter with an optimized RBS	amplified from pDR150 using primers oSW38 and oSW39
DNA fragment	*accDA* coding region	amplified from PY79 genomic DNA using primers oSKH071 and oSK072
DNA fragment	downstream of the *amyE* gene	amplified from PY79 genomic DNA using primers oMD196 and oMD197
Strains	PY79	Wild type strain
Strains	bAB56	*mciZ* ::spec-pHyperSpank-*mciZ*
Strains	bMD834 (Dion, et al., 2019)	*yhdG* ::cat pHyperSpank-*ponA* , *ponA* ::kan, *yvbJ* ::erm-pXyl-*mciZ*
Strains	bMD586	*yhdG* ::cat pHyperSpank-*ponA* , *ponA* ::kan
Strains	bKY42	*ponA* ::kan
Strains	bSW164	*pbpD* ::lox72, *pbpG* ::lox72, *pbpF* ::lox72, *ponA* ::kan, *amyE* ::spec-pSpac-*mciZ*
Strains	bYS19 (Dion, et al., 2019)	*mreB* ::*mreB* -msfGFPsw, *amyE* ::spec
Strains	bSW305	*amyE* ::tet-pXyl-*accDA*
Strains	bMK258	*pbpD* ::erm
Strains	bMK270	*pbpF* ::erm
Strains	bMK260	*pbpG* ::erm
Strains	bMD599 (Dion, et al., 2019)	*ponA* ::kan
Strains	bMK259	*pbpD* ::lox72
Strains	bMK269	*pbpD* ::lox72, *pbpG* ::erm
Strains	bMK273	*pbpD* ::lox72, *pbpG* ::lox72
Strains	bMK275	*pbpD* ::lox72, *pbpG* ::lox72, *pbpF* ::erm
Strains	bMK276	*pbpD* ::lox72, *pbpG* ::lox72, *pbpF* ::lox72
Strains	bSW99 (Hussain, et al., 2018)	*amyE* ::spec-pSpac-*mciZ*
Strains	bSW160	*pbpD* ::lox72, *pbpG* ::lox72, *pbpF* ::lox72, *amyE* ::spec-pSpac-*mciZ*

Table S3. Chemical compositions of *B. subtilis* cell and their refraction increments.

Composition	% of total dry weight (Reference)	Refraction increment [mL/g] (Reference)	Wavelength (nm)
Protein	53 (Bishop et al., 1967)	0.185 (Barer, 1956)	546/589
RNA	18 (Bishop et al., 1967)	0.168-0.194 (Barer, 1956)	546/589
Cell wall	14 (this study)	0.18 (Marquis, 1973)	589
Lipids	5.2 (Bishop et al., 1967)	0.16 (Theisen, 2000) *	not given
DNA	2.6 (Bishop et al., 1967)	0.17-0.2 (Barer, 1956)	546/589
Others **	7.2	0.12-0.14 (Barer and Joseph, 1954) ***	-
Total	100	0.175 ~ 0.182	

* We used the refraction increment of phospholipid.

** We assumed ions and carbohydrates as 'Others'

*** The refraction increment of potassium ion (0.12) and carbohydrates (0.14) were used.

Supplementary videos

video S1. Phase-contrast microscopy of single filamenting cell (bAB56) during vancomycin treatment. Corresponds to Fig 3A.

video S2. MreB-GFP rotation in single cell (bYS19) after vancomycin treatment. Corresponds to Fig 3C-D.

video S3. MreB-GFP rotation in single cell (bYS19) after chloramphenicol treatment. Corresponds to Fig 3G.

video S4. Phase-contrast microscopy of single filamenting cell (bAB56) during cerulenin treatment. Corresponds to Fig 4A, C.

video S5. MreB-GFP rotation in single cell (bYS19) after cerulenin treatment. Corresponds to Fig 4B, C.

References

[1] Michael F Dion, Mrinal Kapoor, Yingjie Sun, Sean Wilson, Joel Ryan, Antoine Vigouroux, Sven Van Teeffelen, Rudolf Oldenbourg, and Ethan C Garner. Bacillus subtilis cell diameter is determined by the opposing actions of two distinct cell wall synthetic systems. *Nature microbiology*, 4(8):1294–1305, 2019.

[2] Gustavo E Schujman, Keum-Hwa Choi, Silvia Altabe, Charles O Rock, and Diego de Mendoza. Response of bacillus subtilis to cerulenin and acquisition of resistance. *Journal of bacteriology*, 183(10):3032–3040, 2001.

[3] Saman Hussain, Carl N Wivagg, Piotr Szwedziak, Felix Wong, Kaitlin Schaefer, Thierry Izoré, Lars D Renner, Matthew J Holmes, Yingjie Sun, Alexandre W Bisson-Filho, et al. Mreb filaments align along greatest principal membrane curvature to orient cell wall synthesis. *Elife*, 7:e32471, 2018.

[4] DG Bishop, L Rutberg, and B Samuelsson. The chemical composition of the cytoplasmic membrane of bacillus subtilis. *European journal of biochemistry*, 2(4):448–453, 1967.

[5] R Barer. Phase contrast and interference microscopy in cytology. *Physical techniques in biological research*, 3:29–90, 1956.

[6] Robert E Marquis. Immersion refractometry of isolated bacterial cell walls. *Journal of bacteriology*, 116(3):1273–1279, 1973.

[7] A Theisen. *Refractive increment data-book for polymer and biomolecular scientists*. Nottingham University Press, 2000.

[8] R Barer and S Joseph. Refractometry of living cells: Part i. basic principles. *Journal of Cell Science*, 3(32):399–423, 1954.
