# Supplementary material for: The role of cell-envelope synthesis for envelope growth and cytoplasmic density in Bacillus subtilis
Source: PNAS Nexus. 2022 Jul 26;1(4):pgac134. doi: 10.1093/pnasnexus/pgac134 (PMC9437589; doi:10.1093/pnasnexus/pgac134)
Supplement: pgac134_Supplemental_Files [file pgac134_supplemental_files.zip › PNASNEXUS-PNASNEXUS-2022-00215-s02.pdf]

Figure	Experiment	Strain	Medium	width * (μm)	S/M * (μm^2/pg)	ρ * (g/mL)	MreB activity or density *	t_{exp} ** (min)	Number of considered cells	t_{MciZ} *** (min)	biological replicates
1B, 1C	snapshot, steady state in S750 + GlcCaa	PY79	S750 + GlcCaa						217		†
1B, S1B	snapshot, steady state in S750 + Glc	PY79	S750 + Glc						315		†
1B	snapshot, steady state in LB	PY79	LB miller						29		†
S1C	timelapse, steady state in S750 + GlcCaa	bAB56	S750 + GlcCaa, 1mM IPTG	0.884	16.34	0.299		0	63	-30	†
1D	timelapse, *ponA* induction	bMD834	S750 + GlcCaa + 1mM IPTG	0.755	17.022	0.331		0	34	-30	1E
1E	snapshot during *ponA* induction	bMD586	S750 + GlcCaa + 1mM IPTG	0.749	16.274	0.346			N = 38, 48, 29, 45, 46, 46, 34, 20, 24, 54<br>when t = -30, 0, 30, 60, 90, 120, 150, 180, 210, 240 min		1D
1G, 1H, S1D	snapshot, aPBP titration (WT)	PY79	S750 + GlcCaa						69		†
1G, 1H, S1D	snapshot, aPBP titration (0 mM)	bMD586	S750 + GlcCaa						94		†
1G, 1H, S1D	snapshot, aPBP titration (0.001mM)	bMD586	S750 + GlcCaa + 0.001 mM IPTG						77		†
1G, 1H, S1D	snapshot, aPBP titration (0.01mM)	bMD586	S750 + GlcCaa + 0.01 mM IPTG						82		†
1G, 1H, S1D	snapshot, aPBP titration (0.1mM)	bMD586	S750 + GlcCaa + 0.1 mM IPTG						89		†
1G, 1H, S1D	snapshot, aPBP titration (1 mM)	bMD586	S750 + GlcCaa + 1 mM IPTG						65		†
1G, 1H, S1D	snapshot, aPBP titration (Δ4)	bSW164 (Δ4)	S750 + GlcCaa						65		†
1G, 1H, S1D	snapshot, aPBP titration (Δ*ponA*)	bKY42	S750 + GlcCaa						75		†
2A, S2A	timelapse, nutrient upshift	bAB56	S750 + Glc, IPTG 1mM (+ 0.4% Caa for upshift)	0.848	16.439	0.313		-30	22	-80	†
2B, S2B	timelapse, nutrient downshift (mild)	bAB56	S750 + GlcCaa, IPTG 1mM (+ 0.4% αMG for downshift)	0.851	15.665	0.313		-30	59	-60	†
2C, S2C, 5A-C	timelapse, nutrient downshift (severe)	PY79	S750 + 2% αMG	0.837	16.499	0.31		0	26		†
S3A	timelapse, hyper-osmotic shock (control)	PY79	S750 + GlcCaa						13		†
S3A	timelapse, hyper-osmotic shock (100 mOsm)	PY79	S750 + GlcCaa + 50 mM NaCl						15		†
S3A	timelapse, hyper-osmotic shock (250 mOsm)	PY79	S750 + GlcCaa + 125 mM NaCl						15		†
S3A	timelapse, hyper-osmotic shock (500 mOsm)	PY79	S750 + GlcCaa + 250 mM NaCl						6		†
S3A	timelapse, hyper-osmotic shock (1 Osm)	PY79	S750 + GlcCaa + 500 mM NaCl						10		†
3A-B, 3D-E, S3B, S3C	timelapse, vancomycin treatment	bAB56	S750 + GlcCaa, IPTG 1mM (+ 50 μg/mL vancomycin)	0.86	15.559	0.313		-30	63	-60	†
3C-E	MreB, vancomycin treatment	bYS19	S750 + GlcCaa (+ 50 μg/mL vancomycin)				1.325 μm^{-1}		N =16-11-10-12-6-8-8-6-7-10-8<br>every 2 min from t = -1~19 min		†
3E, S3D	timelapse, DCS treatment	bAB56	S750 + GlcCaa, IPTG 1mM (+ 10 mM D-cycloserine)	0.891	15.92	0.296		-30	43	-60	†
3E, S3D	timelapse, penicillin treatment	bAB56	S750 + GlcCaa, IPTG 1mM (+ 0.5 mg/mL penicillin G)	0.87	15.688	0.307		-30	46	-60	†
3E, S3D	timelapse, bacitracin treatment	bAB56	S750 + GlcCaa, IPTG 1mM (+ 0.5 mg/mL bacitracin)	0.872	16.077	0.3		-30	23	-60	†
3E, S3D	MreB, DCS treatment	bYS19	S750 + GlcCaa (+ 10 mM/mL D-cycloserine)				1.325 μm^{-1}		N =13-11-8-4-5-6-4-9-9-4-12<br>every 2 min from t = -1~19 min		†
3E, S3D	MreB, bacitracin treatment	bYS19	S750 + GlcCaa (+ 0.5 mg/mL bacitracin)				1.325 μm^{-1}		N =10-6-11-11-11-9-7-8-10-10-12<br>every 2 min from t = -1~19 min		†
3E, S3D	MreB, penicillinG treatment	bYS19	S750 + GlcCaa (+ 0.5 mg/mL penicillin G)				1.325 μm^{-1}		N =18-11-12-12-16-14-17-18-27-10-24<br>every 2 min from t = -1~19 min		†
S3E	timelapse, vancomycin treatment	bSW164 (Δ4)	S750 + GlcCaa, IPTG 1mM (+ 50 μg/mL vancomycin)					-20	32	-60	†
S3F-H	timelapse, hypo-osmotic shock (from 0.6 Osm)	PY79	S750 + GlcCaa (0.6 Osm) → S750 + GlcCaa (0.6, 0.24, 0.04 Osm)<br>adjusted by NaCl addition or by water dilution						N = 16, 21, 22		†
S3F-H	timelapse, hypo-osmotic shock (from 1 Osm)	PY79	S750 + GlcCaa (1 Osm) → S750 + GlcCaa (1, 0.73, 0.65, 0.26 Osm)<br>adjusted by NaCl addition						N = 18, 12, 21, 23		†
3F, 3G	timelapse, chloramphenicol treatment	bAB56	S750 + GlcCaa, IPTG 1mM (+ 100 μg/mL chloramphenicol)	0.837	15.638	0.318		-30	24	-60	†
3G	MreB, chloramphenicol treatment	bYS19	S750 + GlcCaa (+ 100 μg/mL chloramphenicol)				1.325 μm^{-1}		N =9-10-15-9-12-6-10-8-15-7-8-10-7-6-11-13<br>every 2 min during t = -1~29 min		†
4A, 4C	timelapse, cerulenin treatment	bAB56	S750 + GlcCaa, IPTG 1mM (+ 100 μg/mL cerulenin)	0.868	15.285	0.315		-30	35	-60	S4E, S4B
S4B	timelapse, cerulenin treatment	bAB56	S750 + GlcCaa, IPTG 1mM <u>containing</u> 100 μg/mL cerulenin	0.876	15.687	0.312		0	29	-30	4A, S4E
S4C	cerulenin recovery	bAB56	S750 + GlcCaa, IPTG 1mM	0.803	16.554	0.319		0	23	-50	S4D
S4D	cerulenin recovery	PY79	S750 + GlcCaa	0.807	16.116	0.325			N =60-34-38-43-30-24-44-30-49-32<br>when t = -60, -30, 0, 30, 60, 90, 120, 150, 180, 210 min		S4C
4B, 4C	MreB, cerulenin treatment	bYS19	S750 + GlcCaa (+ 100 μg/mL cerulenin)				1.325 μm^{-1}		N =6-5-12-7-10-10-9-6-4-5-6-7-10-12-6-9<br>every 2 min from t = -1~29 min		S4E
S4E	timelapse, cerulenin treatment (carried out at Garner lab)	bYS19	S750 + GlcCaa <u>containing</u> 100 μg/mL cerulenin						38		4A, S4B
S4E	MreB, cerulenin treatment (TIRF-imaging, carried out at Garner lab)	bYS19	S750 + GlcCaa <u>containing</u> 100 μg/mL cerulenin				1.7 μm^{-2}		N =23-31-17-22-16-49<br>when t = 0, 10, 14, 18, 20, 30 min		4B
4D, 4E, S4-2	snapshot during accDA overexpression	bSW305	LB miller + 10 mM xylose	0.845	15.781	0.307			N =10-38-41-55-29-33<br>when t = -15, 0, 30, 60 90, 120 min		†
5A-C	timelapse, control	PY79	S750 + GlcCaa					0	10		†
5A-C	timelapse, vancomycin treatment	PY79	S750 + GlcCaa containing 50 μg/mL vancomycin					0	26		†
5A-C	timelapse, cerulenin treatment	PY79	S750 + GlcCaa containing 100 μg/mL cerulenin					0	18		†
5A-C	timelapse, vancomycin and cerulenin treatment	PY79	S750 + GlcCaa containing +50 μg/mL vancomycin +100 μg/mL cerulenin					0	19		†

* used for normalization
** time of placing cells on agarose pad
*** time of MciZ induction prior to placing cells on agarose pad
biological replicates:
†: confirmed by repeating the same experiment from independent cultures starting from separate colonies or by conducting multiple independent experiments with slight variations (e.g. different osmotic shocks Fig. S3)
Figure numbers: confirmed by very similar experiments as indicated.
