# Supplementary material for: The role of cell-envelope synthesis for envelope growth and cytoplasmic density in Bacillus subtilis
Source: PNAS Nexus. 2022 Jul 26;1(4):pgac134. doi: 10.1093/pnasnexus/pgac134 (PMC9437589; doi:10.1093/pnasnexus/pgac134)
Supplement: pgac134_Supplemental_Files [file pgac134_supplemental_files.zip › PNASNEXUS-PNASNEXUS-2022-00215-s03.pdf]

Name	Sequence
oAB49	GCTGTTTCCCCGCTTACAGC
oAB50	GCTATACGAACGGTAGTTGACCAGTGCTCCCTGGAAGAAGCCCCCTTTTGCTC
oSW40	CAGGGAGCACTGGTC
oSW42	TTCTGCTCCCTCGC
oMD232	GGTAGTTCCTCCTTAAAGCTTAATTGTTATCCGCTCACAAT
oMD234	ATACGAACGGTACTGAGCGAGGGAGCAGAATAATGGATTTCCTTACGCGAAATACG
oAB51	GCGGATAACAATTAAGCTTTAAGGAGGAACTACCGTGAAAGTGCACCGCATGCC
0AB52	AACAAAAATCCATTGACAAACACCA
oMK68	CCTCAGCATTTTCTTCATGGGCTTTG
oMK179	ATACGAACGGTAGTTGACCAGTGCTCCCTGTCTCTTTTCTCCTAAATTCTAGCCATATC
oJM28	TTCTGCTCCCTCGCTCAG
oJM29	CAGGGAGCACTGGTCAAC
oMK180	ATACGAACGGTACTGAGCGAGGGAGCAGAAAGGATCGGAGGGGATATGGAG
oMK181	GCAAATACCGTGACCGAGCTG
oMK173	ATACGAACGGTAGTTGACCAGTGCTCCCTGAACGGGTTCCCCCTTTTTTTATGTTTC
oMK174	GCTTCATCAAATCTCATCTATAATACCC
oMK175	ATACGAACGGTACTGAGCGAGGGAGCAGAAAAATAACCCGGCTCCTCGGAG
oMK176	CGGAGAGCTACGGCTTTATCG
oMK167	ATACGAACGGTAGTTGACCAGTGCTCCCTGGAACTCACCTCGCCTTTCTAAAG
oMK168	CCTTCCGCTTACAGATTATTCATAG
oMK170	ATACGAACGGTACTGAGCGAGGGAGCAGAATGGAATTCGGCGATTTTTTGAACTTTG
oMK171	CAGGTTGCCCTGCACTGTTTTAG
oMD191	TTTGGATGGATTCAGCCCGATTG
oMD108	ACGAACGGTAGTTGACCAGTGCTCCCTGTCTTGACACTCCTTATTTGATTTTTTGAAGAC
oSW38	CATTATACGAACGGTACTGAGCGAGGGAGCAGAAGAATTCGAGCTTGCATG
oSW39	GGTAGTTCCTCCTTAATCG
oSKH071	TTTGAATGGATCGATTAAGGAGGAACTACCTTGTTAAAGGATATATTCACG
oSKH072	TCTTTCGGTAAGTCCCGTCTAGCCTTGCCCTTAGTTTACCCCGATATATT
oMD196	GGGCAAGGCTAGACGGG
oMD197	TCACATACTCGTTTCCAAACGGATC

Name	Description
upstream of the	amplified from PY79 genomic DNA using primers oAB49 and oAB50
spectinomycin-r	amplified from pWX466 using primers oSW40 and oSW42
lacI gene and pI	amplified from pDR111 using primers oMD234 and oMD232
mciZ coding reg	amplified from PY79 genomic DNA using primers oAB51 and oAB52
upstream of the	amplified from PY79 genomic DNA using primers oMK68 and oMK179
erythromycin-re	amplified from pWX469 using primers oJM28 and oJM29
downstream of t	amplified from PY79 genomic DNA using primers oMK180 and oMK181
upstream of the	amplified from PY79 genomic DNA using primers oMK174 and oMK173
downstream of t	amplified from PY79 genomic DNA using primers oMK175 and oMK176
upstream of the	amplified from PY79 genomic DNA using primers oMK168 and oMK167
downstream of t	amplified from PY79 genomic DNA using primers oMK170 and oMK171
upstream of the	amplified from PY79 genomic DNA using primers oMD191 and oMD108
tetracyclin-resist	amplified from pWX470 using primers oSW40 and oSW42
xylR gene and tl	amplified from pDR150 using primers oSW38 and oSW39
accDA coding r	amplified from PY79 genomic DNA using primers oSKH071 and oSK072
downstream of t	amplified from PY79 genomic DNA using primers oMD196 and oMD197

Name	Genotype	Reference
PY79	Wild type strain	-
bAB56	*mciZ* ::spec-pHyperSpank-*mciZ*	this study
bMD834	*yhdG* ::cat pHyperSpank-*ponA* , *ponA* ::kan, *yvbJ* ::erm-pXyl-*mciZ*	*Dion, et al., 2019*
bMD586	*yhdG* ::cat pHyperSpank-*ponA* , *ponA* ::kan	*Dion, et al., 2019*
bKY42	*ponA* ::kan	this study
bSW164	*pbpD* ::lox72, *pbpG* ::lox72, *pbpF* ::lox72, *ponA* ::kan, *amyE* ::spec-pSpac-*mciZ*	this study
bYS19	*mreB* ::*mreB* -msfGFPsw, *amyE* ::spec	*Dion, et al., 2019*
bSW305	*amyE* ::tet-pXyl-*accDA*	this study
bMK258	*pbpD* ::erm	this study
bMK270	*pbpF* ::erm	this study
bMK260	*pbpG* ::erm	this study
bMD599	*ponA* ::kan	*Dion, et al., 2019*
bMK259	*pbpD* ::lox72	this study
bMK269	*pbpD* ::lox72, *pbpG* ::erm	this study
bMK273	*pbpD* ::lox72, *pbpG* ::lox72	this study
bMK275	*pbpD* ::lox72, *pbpG* ::lox72, *pbpF* ::erm	this study
bMK276	*pbpD* ::lox72, *pbpG* ::lox72, *pbpF* ::lox72	this study
bSW99	*amyE* ::spec-pSpac-*mciZ*	*Hussain, et al., 2018*
bSW160	*pbpD* ::lox72, *pbpG* ::lox72, *pbpF* ::lox72, *amyE* ::spec-pSpac-*mciZ*	this study
