# Supplementary material for: The role of cell-envelope synthesis for envelope growth and cytoplasmic density in Bacillus subtilis
Source: PNAS Nexus. 2022 Jul 26;1(4):pgac134. doi: 10.1093/pnasnexus/pgac134 (PMC9437589; doi:10.1093/pnasnexus/pgac134)
Supplement: pgac134_Supplemental_Files [file pgac134_supplemental_files.zip › PNASNEXUS-PNASNEXUS-2022-00215-s04.pdf]

Composition	% of total dry weight (Reference)	Refraction increment [mL/g] (Reference)	Wavelength (nm)
Protein	53 (*Bishop et al., 1967*)	0.185 (*Barer, 1956*)	546/589
RNA	18 (*Bishop et al., 1967*)	0.168-0.194 (*Barer, 1956*)	546/589
Cell wall	14 (this study)	0.18 (*Marquis, 1973*)	589
Lipids	5.2 (*Bishop et al., 1967*)	0.16 (*Theisen, 2000*) *	not given
DNA	2.6 (*Bishop et al., 1967*)	0.17-0.2 (*Barer, 1956*)	546/589
Others **	7.2	0.12-0.14 (*Barer and Joseph, 1954*) ***	-
Total	100	0.175 ~ 0.182	

* We used the refraction increment of phospholipid.

** We assumed ions and carbohydrates as 'Others'

*** The refraction increment of potassium ion (0.12) and carbohydrates (0.14) were used.
